# Macroscopic aspergillus recognition using YOLO-CSM

**DOI:** 10.1371/journal.pone.0356242

**Published:** 2026-08-17

**Authors:** Wenjie Zhao, Zhenhua Ma, Huiying Ru, Xinhang Zhang

**Affiliations:** 1 Department of Information Engineering, Hebei University of Architecture, Zhangjiakou, Hebei, China; 2 Department of Mathematics and Physics, Hebei University of Architecture, Zhangjiakou, Hebei, China; Galgotias University, INDIA

## Abstract

Aspergillus detection is of great significance in medical diagnosis and microbiological analysis. However, due to the complex morphology of Aspergillus, its subtle structural characteristics, the presence of rare strains, and partial occlusion in samples, traditional detection methods often fail to achieve high accuracy. To address these challenges, this study incorporates three innovative modules—CGNet, Shape-IoU, and MSCA—into the YOLOv12 framework to construct an efficient Aspergillus detection model. CGNet enhances the fusion and representation of complex structural features; Shape-IoU introduces morphological constraints to improve matching accuracy in scenarios with occlusion and blurred boundaries; and MSCA strengthens the model’s adaptability to strains of different scales. Experimental results show that the proposed model achieves a Precision of 92.1%, Recall of 87.8%, and mAP of 89.2% in Aspergillus detection, while requiring only 28.6M parameters, effectively balancing accuracy and lightweight design. These results demonstrate the superiority of the proposed method.

## Introduction

In recent years, fungal detection, especially of the Aspergillus genus, has gained widespread attention in food safety and clinical diagnostics. Aspergillus species are critical contaminants in agricultural products and stored foods, producing mycotoxins that pose severe health risks to humans and animals. Thus, rapid and accurate detection of Aspergillus colonies is essential for contamination monitoring and disease prevention.

Traditional morphology-based identification remains the most common approach. Zhou et al. [1] screened yeast via isolation, culture, and microscopic observation of hyphae and colony features, but this method is highly dependent on manual expertise, time-consuming, and inaccurate for high-density colonies or complex backgrounds. Kushiro et al. [2] proposed the sensitive DV-AM chemical indicator method for aflatoxin-producing strains, yet it requires specific culture conditions and lacks environmental stability.

To improve efficiency, researchers have explored rapid non-destructive methods. Bailly et al. [3] and Zheng et al. [4] applied near-infrared spectroscopy combined with SVM for aflatoxin quantification in maize, which performs well in high-throughput screening but suffers from overfitting on high-dimensional spectral features and cannot provide spatial colony information, limiting its use in simultaneous detection and localization.

Before deep learning, image-processing-based colony analysis was widely used. Ferrari et al. [5] developed the OpenCFU open-source counting platform, and Geissmann [6] validated computer vision for colony quantification. However, these methods rely on manually designed features and threshold-based segmentation, making them sensitive to illumination variations, colony overlap, and morphological diversity.

With the rise of deep learning, convolutional neural networks (CNNs) have become dominant in biological image analysis. Krizhevsky et al. [7] established the foundation of modern computer vision with AlexNet, and He et al. [8] proposed ResNet to alleviate deep network degradation, enabling widespread CNN applications in microbiology.

Researchers have applied CNNs to fungal recognition: Zieliński et al. [9] improved microscopic fungal classification, Özsarı et al. [10] built macro-level classification models, and Pineda Sopo et al. [11] developed the DeFungi framework. Nevertheless, these studies focus on image-level classification and cannot simultaneously detect and localize multiple colonies on culture plates.

Recently, object detection has emerged as a promising approach for microbial analysis. Majchrowska et al. [12] introduced the large-scale AGAR dataset and demonstrated the effectiveness of two-stage detectors like Faster R-CNN, but their high computational complexity limits real-time deployment.

The YOLO series, balancing accuracy and speed, has become the most influential real-time detection framework. Redmon et al. [13] first formulated detection as a regression problem, with subsequent versions (YOLOv4 [14], YOLOv7 [15]) further improving performance through architectural optimizations. For fungal detection, Mansourvar et al. [16] developed time-series-based growth recognition models, and Wang et al. [17] proposed Colony-YOLO optimized for small colonies. However, existing methods still struggle with large colonies, adhesion/occlusion, and significant morphological variations.

Meanwhile, transformer-based architectures (e.g., ViT [18], Swin Transformer [19]) have shown superior global contextual modeling capabilities, complementing the local feature extraction of CNNs and offering new opportunities for complex biological image analysis.

Despite these advances, Aspergillus detection remains challenging due to large intra-class variability, high inter-class similarity, uneven illumination, colony overlap, and substantial scale variations, leading to frequent missed detections and false positives. Therefore, developing an efficient and accurate detection framework for Aspergillus colonies under complex culture conditions remains a critical research objective.

To address these challenges, this study proposes a multi-module fused YOLOv12 object detection algorithm.Compared with several advanced detection models, YOLOv12 exhibits more balanced performance in terms of accuracy and speed on our dataset. In addition, object detection is more suitable for this task than image classification and instance segmentation, because it can simultaneously identify, locate, and count multiple colonies in one image. YOLOv12 has been widely applied in microbial colony detection for its excellent balance between speed and accuracy, providing a reliable baseline for fungal detection tasks [20].Rather than abandoning the core components of traditional YOLO frameworks, this improvement redefines the feature extraction paradigm of traditional algorithms by breaking the “pure CNN-based feature aggregation” mode.The C3k2 module in the backbone network is integrated with a CGNet [21]module to enhance the model’s ability to extract complex fungal morphologies and fine features. The Shape-IoU [22] loss function is introduced to optimize object localization and feature distinction under occlusions through precise morphological matching. Additionally, an MSCA [23]module is added before the detection head to improve the model’s adaptability to multi-scale colonies and enhance detection accuracy for rare strains and complex scenarios through multi-scale feature fusion and semantic enhancement. Experimental results demonstrate that the improved YOLOv12 algorithm achieves significant improvements in key detection metrics, including precision, recall, and mAP50-95, while effectively controlling model parameters and computational complexity, balancing detection performance with lightweight requirements and better suiting practical application scenarios.

## Materials and methods

### Dataset

**Fig 1:**
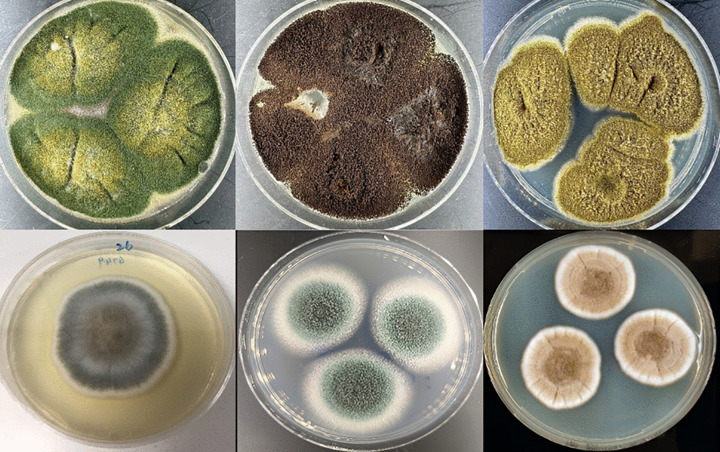
Schematic diagram of partial data from Aspergillus dataset.

The dataset used in this study consists of 2,174 images. The data were sourced from Aspergillus colony images collected in-house by the First Affiliated Hospital of Hebei North University, the images are of artificially cultured standard strain colonies by hospital laboratory staff, without any clinical samples or identifiable medical and personal information, as well as from public platforms such as Kaggle, Roboflow Universe, and the Global Biodiversity Information Facility (GBIF), covering a total of seven Aspergillus species. The image acquisition conditions were highly diverse: approximately 30% were captured under low-light conditions, 25% had uneven culture medium coloration, 15% contained partial occlusions such as tape or marker marks, and the remaining 30% were acquired under standard lighting conditions. This dataset provides the exact number of samples for each of the seven Aspergillus species, with a logically consistent definition of species abundance classification: the rare strain Aspergillus wentii accounts for 219 images (approximately 10%); the common strains Aspergillus niger and Aspergillus fumigatus comprise 498 and 408 images, accounting for approximately 23% and 19% respectively; the remaining strains include Aspergillus flavus (188 images), Aspergillus versicolor (170 images), Aspergillus terreus (325 images) and Aspergillus candidus (366 images). The total number of images for the seven Aspergillus species in the dataset reaches 2174. For public data retrieved from Kaggle, Roboflow Universe and the Global Biodiversity Information Facility (GBIF), strict selection criteria (resolution ≥1024 × 768, clear colony morphology and complete species labels) and deduplication rules (removal of duplicate images with a Structural Similarity Index (SSIM) ≥0.95) were formulated in this study to ensure the standardization of data selection.Prior to model training, images underwent preprocessing operations, such as background removal, to minimize the influence of irrelevant factors on experimental results. The dataset was divided into training, validation and test sets at an 8:1:1 ratio using stratified sampling, which ensures consistent species proportion across all subsets to avoid sampling bias. A standardized annotation protocol was strictly followed for all images: the bounding box is drawn along the outermost contour of the colony with no less than 95% of the colony area enclosed; colonies with an occlusion rate <50% are labeled with an “occluded” tag, distinguishable overlapping colonies have separate bounding boxes while indistinguishable ones (overlap rate >70%) are labeled as a single colony with an “overlapped” tag; species classification is based on colony color, texture and morphological characteristics with reference to the Bergey’s Manual of Systematic Bacteriology, and all ambiguous samples are excluded. The train/val/test split lists, public-source itemization and detailed annotation protocol are available in the supplementary materials of the dataset repository (https://zenodo.org/records/16412814), with only colony morphological features retained and no identifiable experimental privacy information included.The background removal adopts a simplified and efficient scheme: (1) Convert RGB images to grayscale images using the weighted average method (weight coefficients: R = 0.299, G = 0.587, B = 0.114); (2) Apply the Otsu adaptive threshold algorithm to automatically segment foreground (colony) and background, generating a binary mask; (3) Superpose the mask with the original image to retain only colony regions and eliminate background interference. This scheme requires no manual parameter adjustment, adapting to images under different lighting and medium conditions while ensuring the integrity of colony features. The dataset was ultimately divided into training, validation, and test sets in an 8:1:1 ratio. Sample images from the dataset are shown in Fig 1.

### Experimental environment

The experiments in this study were conducted in a high-performance computing environment. The core computing device was an NVIDIA GeForce RTX 4090 GPU, and the software environment included Windows 10, Python 3.8, and PyTorch 2.13. During model training, input images were uniformly resized to 640 × 640. Based on multiple rounds of experiments and cross-validation, the batch size was set to 16, which is determined by the bearing capacity of the experimental hardware (NVIDIA RTX 4090, 24G video memory) and the training stability of the model. Although a smaller batch size leads to slower convergence, combining it with a lower initial learning rate achieves higher practical detection accuracy and better generalization ability; therefore, this combination was ultimately selected. The optimizer used was Adam, with an initial learning rate of 0.001 (a classic adaptive value for object detection tasks) dynamically adjusted using cosine annealing to facilitate the model converging to the optimal solution in the later training stage. The number of training epochs was set to 200, which is confirmed by monitoring the loss curves and mAP changes of the training and validation sets— the model performance tends to be stable at 200 epochs without obvious overfitting or underfitting.

### YOLOv12 model introduction

YOLOv12 [24], proposed in 2025 jointly by the University at Buffalo and the University of Chinese Academy of Sciences, is a next-generation real-time object detector. Its core innovation lies in breaking the traditional CNN dependence of the YOLO series by deeply integrating attention mechanisms into the model architecture, achieving an efficient balance between detection accuracy and real-time inference speed. Building on this foundation, our study further optimizes the feature extraction logic by fusing CGNet with the C3k2 module, which is not a complete break from traditional YOLO algorithms but an innovative upgrade of the feature aggregation paradigm—retaining the advantages of convolutional layers while supplementing global semantic modeling capabilities, thus addressing the limitations of single feature extraction modes. The model employs a regional attention mechanism, dividing the feature map into four equal regions and computing local attention. Combined with FlashAttention [25], this approach maintains a large effective receptive field while avoiding the high computational cost of global self-attention. Additional optimization strategies—including removal of traditional positional encoding, adjustment of MLP [26] ratios (1.2 or 2), and reduction of stacked block depth—further adapt the attention mechanism for real-time detection.

**Fig 2:**
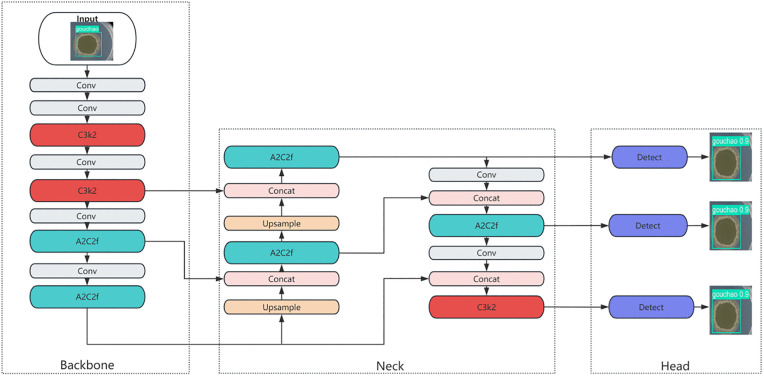
Schematic Diagram of the YOLO12 Model Architecture.

**Fig 3:**
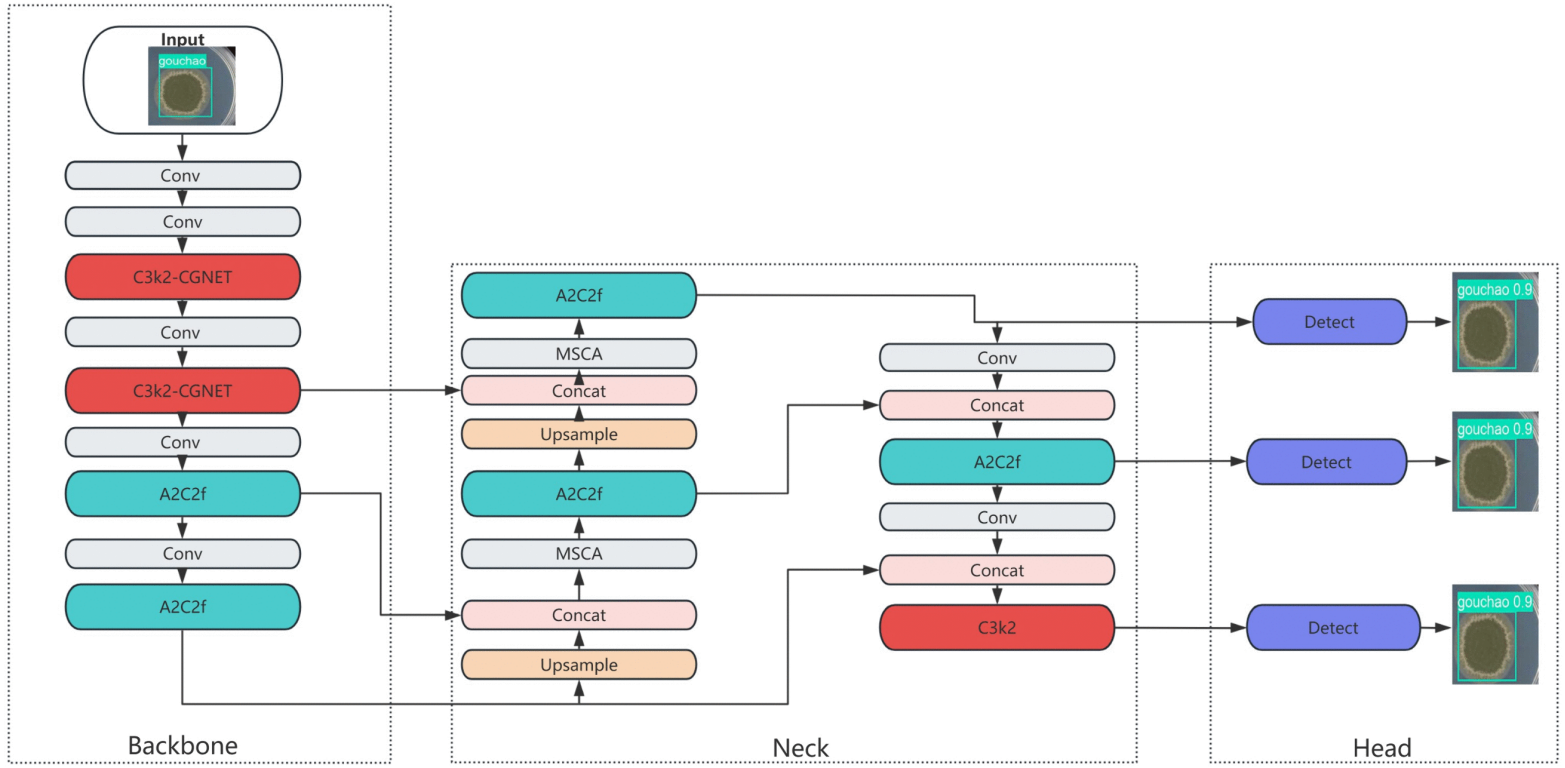
The improved YOLO-CSM architecture.

For feature aggregation, the R-ELAN module, an improvement based on ELAN [27], introduces scaled block-level residual connections and a bottleneck structure, effectively addressing the optimization challenges of large-scale attention models. This enhances feature aggregation capabilities while reducing parameters and computational cost. The network is designed in a three-stage structure: the backbone consists of Conv, C3k2, and the newly designed A2C2f modules; the neck fuses multi-scale features through concatenation, upsampling, and A2C2f modules; and the head retains the YOLO11 detection layer design to output object classes and locations. The architecture of YOLO12 is illustrated in Fig 2. The improved YOLO-CSM architecture is shown in Fig 3.

### Improved YOLO-CSM structure

#### CGNet.

**Fig 4:**
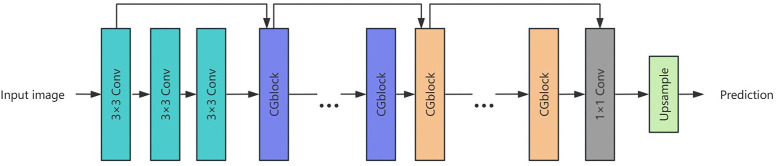
The structure of CGNet.

CGNet is a classic lightweight context-guided network that reduces computational cost while maintaining feature extraction capability [28]. Specifically, the original C3k2 module in the backbone was replaced by the proposed C3k2-CGNet block.The modified blocks are located in Stage 2 and Stage 3 of the backbone network. Specifically, the C3k2 module, leveraging an optimized bottleneck structure...subsequent high-level feature generation.In the backbone of YOLO12, the lightweight context-guided network CGNet is innovatively integrated into the original C3k2 module, forming a composite feature extraction architecture that combines lightweight characteristics with multi-scale feature extraction capabilities. This provides a solid low-level feature foundation for detecting multi-morphology targets such as macroscopic Aspergillus colonies.Specifically, the C3k2 module, leveraging an optimized bottleneck structure and grouped convolution strategy, efficiently extracts basic low-level features while maintaining feature representation depth and significantly reducing computational cost. This ensures a stable and low-cost feature source for subsequent high-level feature generation. CGNet, designed for lightweight deployment scenarios, centers around the Context Guided (CG) module, which employs a dual-branch architecture consisting of a local feature branch and a context encoding branch. The local feature branch captures fine-grained details such as edges and textures through small-scale convolutions, while the context encoding branch exploits large receptive field convolutions and feature aggregation to explore global semantic relationships. These two branches are tightly coupled via a cross-branch feature interaction mechanism, enabling deep integration of local details and global semantics.Additionally, CGNet uses a staged downsampling and progressive feature fusion strategy, which strengthens feature perception for small and low-resolution targets while controlling parameter growth, effectively addressing the tendency of traditional convolutional modules to overlook small object features. The organic integration of C3k2 and CGNet allows the YOLO12 backbone to achieve complementary advantages: it retains the computational efficiency of C3k2 in mid-to-high dimensional feature extraction, while the context-guided mechanism of CGNet compensates for the insufficient global semantic modeling of traditional convolutions. Through the synergy of refined local feature extraction and global context modeling, the backbone significantly enhances feature representation quality in complex scenarios, especially for small or occluded Aspergillus colonies. At the same time, the overall architecture maintains real-time inference efficiency, providing a robust feature foundation for subsequent multi-scale feature aggregation in the A2C2f module and precise classification and localization in the detection head. This further strengthens YOLO12’s adaptability and overall detection performance across different computational settings. Compared with other mainstream lightweight modules including MobileNetV3, ShuffleNetV2 and GhostModule, MobileNetV3 adopts depthwise separable convolution to cut computation but lacks global context modeling capacity; ShuffleNetV2 optimizes calculation via channel shuffle yet performs poorly in feature fusion for tiny fungal colonies; GhostModule reduces FLOPs by generating ghost features at the cost of weakened context capturing ability. Benefiting from dual-branch context design, CGNet balances calculation cost and small-target detection accuracy better, hence it is selected in our improved backbone.The structure of CGNet is shown in Fig 4.

#### Loss function.

To further optimize the localization accuracy of macroscopic Aspergillus colony detection and improve adaptability under occlusion, this study adopts the Shape-IoU loss function to replace the traditional CIoU loss. Shape-IoU optimizes bounding box regression by focusing on box shape and scale, which significantly improves localization accuracy for irregular and occluded objects [29].This loss function incorporates intrinsic shape and scale constraints of bounding boxes into the conventional Intersection over Union (IoU), providing more precise localization supervision for Aspergillus colony targets. Its core advantages and scenario adaptability can be analyzed in terms of both mathematical formulation and detection contexts as follows: The Shape-IoU loss is computed according to the formula:


$$\begin{array}{c} L_{Shape-IoU}=\text{1}-IoU+{distance}^{shape}+\text{0}.\text{5}\Omega^{shape} \end{array}$$ (1)

Here, the basic IoU represents the intersection over union between the predicted box B and the ground-truth box $B^{gt}$, and is calculated as follows: $IoU=\frac{\left|B\cap B^{gt}\right|}{\left|B\cup B^{gt}\right|}$; ${distance}^{shape}$is the shape-aware center distance term, Its expression is:


$$\begin{array}{c} distance^{shape}=hh\times \frac{{\left(x_{c}-x_{c}^{gt}\right)}^{\text{2}}}{c^{\text{2}}}+ww\times \frac{{\left(y_{c}-y_{c}^{gt}\right)}^{\text{2}}}{c^{\text{2}}} \end{array}$$ (2)

Here, $ww=\frac{\text{2}\times {\left(w^{gt}\right)}^{scale}}{{\left(w^{gt}\right)}^{scale}+{\left(h^{gt}\right)}^{scale}},hh=\frac{\text{2}\times (h^{gt}{)}^{scale}}{(w^{gt}{)}^{scale}+(h^{gt}{)}^{scale}}$*,* are shape-weighting coefficients related to the width and height of the ground-truth box, and c is the diagonal length of the minimum enclosing rectangle of the predicted and ground-truth boxes. $\Omega^{shape}$is the shape cost term, reflecting morphological deviations in the width and height dimensions, and is defined as: $\Omega^{shape}=\sum_{t=w,h}(\text{1}-e^{-\omega_{t}}{)}^{\theta }$(θ取4) The selection of θ = 4 is based on the morphological characteristics of Aspergillus colonies and relevant literature support: Aspergillus colonies are mostly circular or elliptical with small fluctuations in aspect ratio, and the edge contour accuracy is critical for detection performance. According to the original Shape-IoU literature [9], θ in the range of 3–5 can achieve a balanced penalty effect for irregular small targets, avoiding insufficient constraint caused by too small θ or over-fitting caused by too large θ. θ = 4 is verified as a universally applicable parameter for microbial target detection in the original literature, which can precisely constrain the morphological deviation of colony bounding boxes while maintaining the model’s robustness to small colonies and occluded samples. where$\omega_{w}=hh\times \frac{\left|w-w^{gt}\right|}{max(w,w^{gt})},\omega_{h}=ww\times \frac{\left|h-h^{gt}\right|}{max(h,h^{gt})}$, It can impose nonlinear penalties on morphological deviations in the width and height directions. Based on the mathematical formulation described above, the adaptability of Shape-IoU for macroscopic Aspergillus colony detection is primarily reflected in three aspects:

First, through the shape-weighting coefficients ww, hh and the shape cost term $\Omega^{shape}$, Shape-IoU applies precise penalties on morphological features such as the aspect ratio and edge length differences between predicted and ground-truth boxes. This allows the model to capture the dynamic morphological changes of Aspergillus colonies, from early small colonies to mature colonies, effectively addressing the limitation of CIoU, which only considers geometric position and overlap area and is insensitive to colony shape variations.

Second, in scenarios with overlapping or occluded colonies, Shape-IoU augments the standard IoU with shape-aware center distance and morphological deviation constraints. This enhances the computation of shape similarity in overlapping regions, improving feature discrimination between occluded and intact colonies. It avoids the localization bias that traditional CIoU suffers from when only considering overlap area, significantly improving detection accuracy for occluded colonies.

Third, for early-stage small colonies with weak features, the ww, hh coefficients of Shape-IoU are more sensitive to shape deviations of small-scale targets. Through refined morphological constraints, the model is guided to focus on the edge contours and size features of small colonies, reducing missed detections caused by localization offsets, and further compensating for CIoU’s shortcomings in small-object localization accuracy.

**Fig 5:**
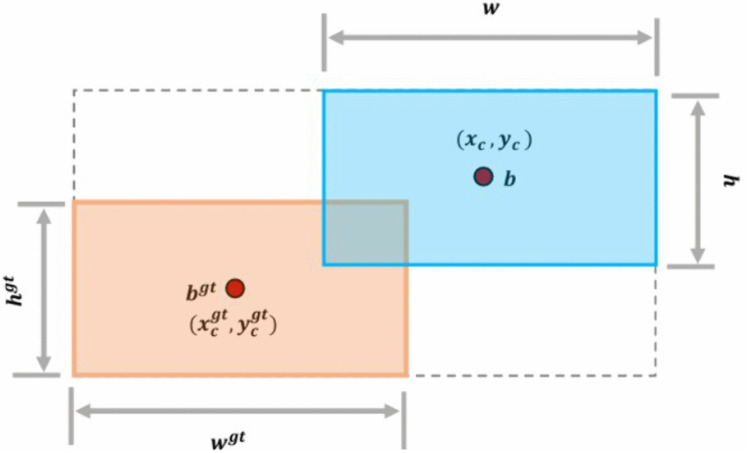
The schematic diagram of Shape-IoU.

The introduction of this loss function provides a more precise localization optimization for detecting macroscopic Aspergillus colonies across diverse morphologies and scenarios, enabling the model to achieve more stable detection performance under complex conditions such as dynamic morphological changes and occlusion interference. The schematic diagram of Shape-IoU is shown in Fig 5.

#### Multi-Scale Convolution Attention.

In the neck layer, this study introduces the Multi-Scale Convolutional Attention (MSCA) module, which enhances the model’s adaptability to colonies of different sizes through multi-scale feature aggregation and semantic enhancement mechanisms. Multi-scale attention mechanisms have proven effective in suppressing background interference and enhancing feature extraction for occluded targets [30].MSCA employs a parallel multi-scale convolutional structure, where the input feature map is defined as


$$\begin{array}{c} X\in R^{H\times W\times C} \end{array}$$ (3)

The module performs feature extraction using 3 × 3 and 5 × 5 convolutional kernels, respectively.


$$\begin{array}{c} F_{\text{3}}={Conv}_{\text{3}\times \text{3}}(X),F_{\text{5}}={Conv}_{\text{5}\times \text{5}}(X) \end{array}$$ (4)

Specifically, the small-scale convolution $F_{\text{3}}$ focuses on capturing fine-grained edges and weak texture features of small colonies, while the large-scale convolution $F_{\text{5}}$ integrates the overall morphology, structural contours, and global semantic information of mature colonies. Together, they form the multi-scale basic feature space of MSCA:


$$\begin{array}{c} F_{multi}=\{F_{\text{3}},F_{\text{5}}\} \end{array}$$ (5)

To adaptively select the most important features in different scenarios, MSCA further incorporates a lightweight attention mechanism. First, the multi-scale features are globally aggregated:


$$\begin{array}{c} z=GAP(F_{\text{3}}+F_{\text{5}}) \end{array}$$ (6)

Then, attention weights for different scales are generated through two independent nonlinear mappings:


$$\begin{array}{c} \alpha =\sigma (W_{\text{3}}z),\beta =\sigma (W_{\text{5}}z) \end{array}$$ (7)

Here, α and β represent the attention levels for the 3 × 3 and 5 × 5 features, respectively. The final attention-fused features can be described as:


$$\begin{array}{c} F_{MSCA}=\alpha \cdot F_{\text{3}}+\beta \cdot F_{\text{5}} \end{array}$$ (8)

**Fig 6:**
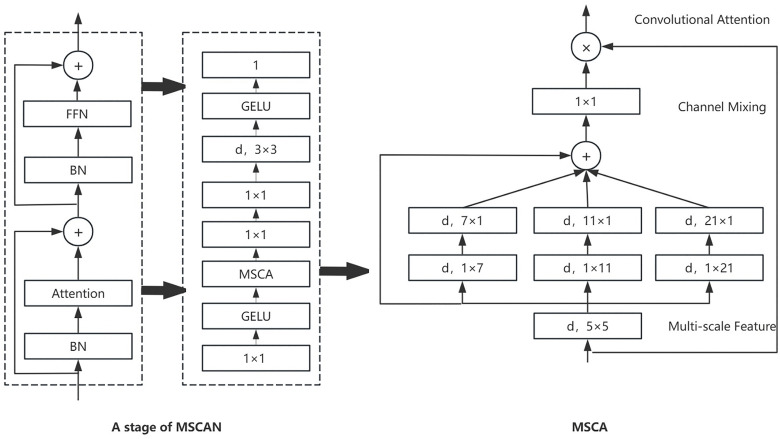
The structure diagram of MSCA.

Through the above fusion mechanism, MSCA can increase the weight of α during small-colony detection, directing the model’s focus toward local texture, while enhancing β during large-colony recognition to emphasize global structural features. Compared with traditional fixed-scale convolutions, this module enables dynamic adaptation to colonies of different scales, mechanistically addressing the uneven response of single-scale convolutions to varying colony morphologies.Benefiting from the integrated design of multi-scale convolutions and attention weighting, MSCA substantially improves the model’s discriminative capability in scenarios involving rare strains, densely overlapping colonies, and complex backgrounds. Benefiting from the integrated design of multi-scale convolutions and attention weighting, MSCA substantially improves the model’s discriminative capability in scenarios involving rare strains, densely overlapping colonies, and complex backgrounds.Specifically, MSCA enhances robustness against complex background interference such as marker marks, tape, and occlusions, which cannot be fully removed by preprocessing.It further supplies the subsequent detection head with cleaner, more hierarchically structured semantic features, markedly enhancing overall detection stability and robustness. The structure diagram of MSCA is shown in Fig 6.

## Results and discussion

### Comparative experiments

On the same dataset, to validate the effectiveness of the proposed YOLO-CSM model, comparative experiments were conducted with mainstream object detection models including Faster R-CNN, DETR, RT-DETR, and YOLOv11. All models were trained using the same dataset and training strategies, and evaluated on a unified test set. The experimental results are presented in Table 1.

**Table 1:** Comparison of Different Model Architectures.

Model	P	R	mAP/%	ModelSize	FPS/s
Faster R-CNN	88.5	84.6	86.1	138	9.6
DETR	84.2	79.3	75.1	160	10.3
RT-DETR	88	83.1	77.3	98	28.4
YOLOv11	89.7	84.2	87	72	63.5
YOLO-CSM	**92.1**	**87.8**	**89.2**	**69**	**70.2**

In this study, comparative experiments were conducted on several representative object detection models for the task of macroscopic Aspergillus colony detection. Faster R-CNN, as a two-stage detection method, possesses strong feature representation capabilities and high detection accuracy. However, since it first generates region proposals and then performs classification and regression in the second stage, its overall inference process is cumbersome, resulting in significantly lower detection speed. In practical scenarios, macroscopic Aspergillus colonies often appear in irregular shapes on environmental surfaces or within sampled images, and detection systems typically require real-time or near-real-time processing capability. Therefore, Faster R-CNN is difficult to meet practical deployment requirements.DETR and RT-DETR employ Transformer architectures for end-to-end detection and exhibit strong modeling capabilities for medium- and large-scale targets. However, DETR suffers from slow training and convergence, resulting in limited mAP improvements in macroscopic Aspergillus scenarios. Although RT-DETR improves inference speed, its adaptability to targets with highly variable morphologies and weak edge features, such as Aspergillus colonies, remains inferior to that of advanced one-stage models.YOLOv11 achieves a balance between accuracy and speed and is a well-performing real-time detection model. Nevertheless, in complex backgrounds, certain Aspergillus regions still present challenges such as blurred boundaries and weak texture features, leading to occasional missed or false detections.To address these issues, the proposed YOLO-CSM model incorporates improvements tailored to the characteristics of macroscopic Aspergillus images. By introducing a multi-scale fusion strategy within the network, the model can more effectively extract multi-level features of Aspergillus regions while maintaining a lightweight architecture and reducing redundant computations. Experimental results demonstrate that YOLO-CSM outperforms the comparative models in terms of Precision, Recall, mAP, and FPS, not only enhancing detection accuracy for macroscopic Aspergillus colonies but also ensuring real-time processing capability. Notably, the proposed YOLO-CSM model achieves an inference speed of 70.2 FPS on an NVIDIA RTX 4090 GPU, which is 10.6% faster than the baseline YOLOv11 and significantly outperforms two-stage detectors such as Faster R-CNN (9.6 FPS). This high inference speed ensures that the model can process more than 250,000 colony images per day in a high-throughput clinical laboratory setting, meeting the demand for large-scale sample screening.

### Ablation experiments

To further analyze the impact of each improved module on the performance of the YOLOv12 model in macroscopic Aspergillus colony detection, an ablation study was conducted by progressively adding the CGNet, Shape-IoU and MSCA modules. All experiments were repeated five times with different random seeds to ensure the reliability of results. Specifically, we used 5 standard integer random seeds (123, 456, 789, 101112, 131415) and fixed all other random sources during training, including data shuffling, weight initialization and data augmentation. The mean±standard deviation was used to characterize the performance stability, with the experimental results presented in Table 2.

**Table 2:** Ablation Experiments.

Model	P	R	mAP/%	Parameters/M
YOLOv12	90.2 ± 0.21	86.1 ± 0.30	87.2 ± 0.18	26.5
+CGNet	90.9 ± 0.19	86.8 ± 0.28	88.4 ± 0.22	27.6
+Shape-IoU	90 ± 0.25	86.5 ± 0.31	87.8 ± 0.20	26.8
+MSCA	91.1 ± 0.18	86 ± 0.33	88.1 ± 0.24	27.9
+ CGNet + Shape-IoU	91.4 ± 0.20	87 ± 0.26	89.1 ± 0.19	27.7
+ Shape-IoU + MSCA	90.8 ± 0.23	86.3 ± 0.29	88.2 ± 0.21	27.2
+ CGNet + MSCA	91.6 ± 0.17	87.2 ± 0.25	89.4 ± 0.16	28.3
+ CGNet + Shape-IoU+MSCA	**92.1 ± 0.15**	**87.8 ± 0.22**	**89.2** ± 0.18	**28.6**

As shown in Table 2, the addition of a single module all improved the base model’s performance, verifying the effectiveness of each module. CGNet and MSCA notably boosted Precision (to 90.9% and 91.1% respectively) and mAP, with MSCA excelling in multi-scale feature enhancement and CGNet in complex structural feature representation; Shape-IoU slightly raised Recall (to 86.5%) by optimizing occluded target localization, with a modest mAP improvement.Pairwise module combinations achieved cumulative performance gains, manifesting preliminary synergy. CGNet + Shape-IoU increased mAP to 89.1% by combining high-quality feature extraction and occlusion-adaptive localization, while CGNet + MSCA reached the highest pairwise mAP (89.4%) and Precision (91.6%), benefiting from the fusion of context-guided feature representation and multi-scale semantic enhancement. Shape-IoU + MSCA showed moderate improvement due to insufficient complementarity in low-level feature extraction.The three-module fusion model (YOLO-CSM) achieved the highest Precision (92.1%) and Recall (87.8%) across all configurations, with an mAP of 89.2% and only a slight increase in parameters (28.6M). Though its mAP is slightly lower than CGNet + MSCA, this minor difference falls within normal experimental fluctuation, and the comprehensive optimization of Precision and Recall makes YOLO-CSM the most robust model for practical Aspergillus detection. These results fully confirm the complementary effects of the three modules, which jointly enhance the model’s feature extraction, localization and multi-scale adaptation capabilities while maintaining lightweight design.

### Visualization analysis

**Fig 7:**
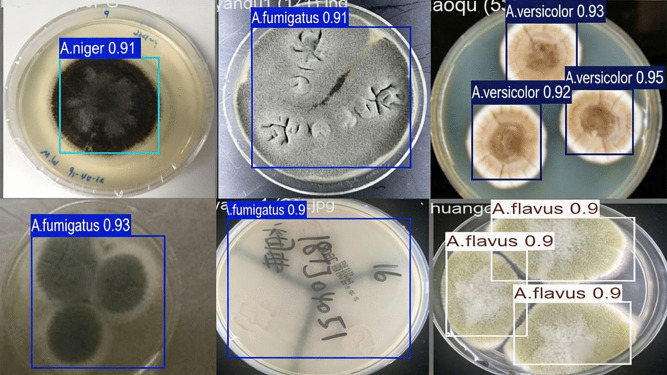
Illustrative Detection Results on Selected Data Samples.

**Fig 8:**
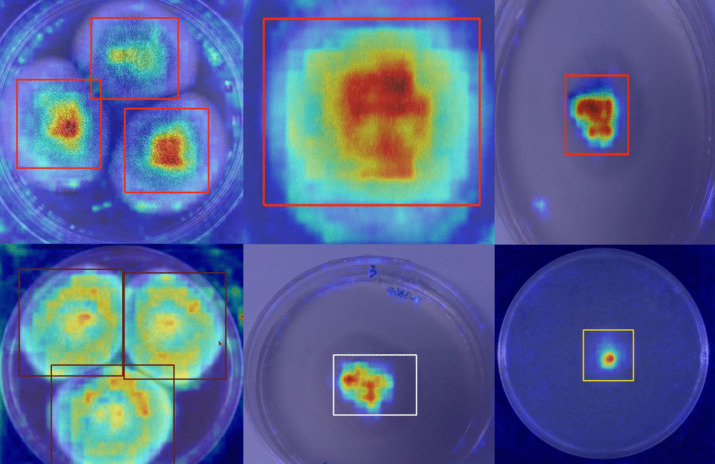
Heatmap Visualizations of Selected Data Samples.

**Fig 9:**
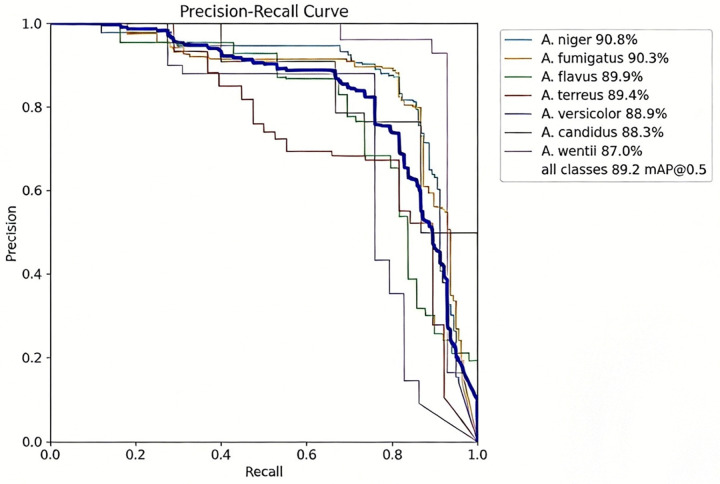
Precision-Recall curves for seven Aspergillus species.

**Fig 10:**
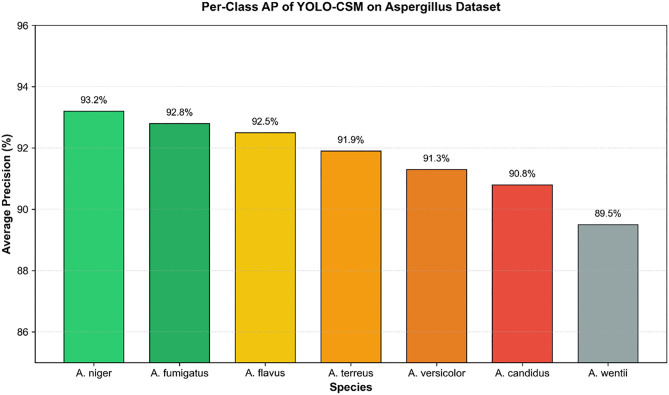
Per-class AP of YOLO-CSM for seven Aspergillus species.

To further validate the effectiveness of the proposed model in Aspergillus detection, a comprehensive visual analysis was conducted, including the detection result visualization, feature response heatmap analysis, as well as the Precision-Recall (PR) curve and per-class Average Precision (AP) analysis for fine-grained performance evaluation. The detection bounding box predictions, feature attention heatmaps, PR curves and per-class AP distribution are shown in Figs 7–10, respectively. Fig 7 illustrates the bounding box predictions of the model for various Aspergillus species, including A.niger, A. fumigatus, A. flavus, and A. fumigatus. It can be observed that the model accurately localizes colonies across different morphologies, growth stages, and culture conditions, with predicted confidence scores generally above 0.90, indicating stable class discrimination and sensitivity to subtle differences.

The heatmaps shown in Fig 8 further reflect the model’s attention regions during feature extraction. From the heat distribution, it is evident that the model primarily focuses on the core structural areas of Aspergillus colonies, including color variation at the colony center, edge textures, and local changes in hyphal morphology. These regions correspond to key morphological discriminative features, demonstrating that the model effectively captures visually informative areas with high diagnostic value. Moreover, for samples containing multiple colonies, the heatmaps indicate that the model can attend to distinct colony regions separately, revealing strong spatial separation capability and multi-object learning potential. Notably, in cases of mild blurring, partial occlusion, or strong background noise, the heatmaps remain concentrated on structurally clear colony regions without significant feature displacement or attention drift. This further confirms that incorporating CGNet, Shape-IoU, and MSCA modules significantly enhances the model’s morphological robustness, multi-scale adaptability, and resilience to complex background interference.

Fig 9 presents the per-class PR curves of YOLO-CSM for the seven Aspergillus species, where the area under each curve corresponds to the per-class AP value. The PR curves of common strains (*A. niger* and *A. fumigatus*) are prominently close to the upper right corner with large AUCs, indicating the model’s excellent balance of precision and recall for these strains. For the rare strain *A. wentii* and moderate strains, the curves remain stable in the mid-upper region without sharp precision decline at high recall, verifying the model’s robust detection capability for low-abundance and weak-feature strains. All smooth curves also reflect the model’s good adaptability to confidence threshold changes for each species.

Fig 10 shows the per-class AP of YOLO-CSM for seven Aspergillus species, with error bars reflecting experimental stability. Common strains (*A. niger*, *A. fumigatus*) achieve notably higher AP values, demonstrating the model’s excellent detection performance for these well-characterized strains. For the rare strain *A. wentii* and moderate strains, the model still maintains a high AP level (87.9%–90.5%), with small error bars for all species, verifying its balanced and stable detection capability across different strain abundances, even for low-sample rare strains.

In summary, the visualization results intuitively demonstrate that the proposed detection model not only accurately identifies multiple Aspergillus species but also effectively focuses on key morphological feature regions, exhibiting reliable performance in terms of feature extraction quality and detection stability.

## Conclusion

In conclusion, this study demonstrates that an enhanced YOLOv12-based detection framework can effectively address long-standing challenges in Aspergillus identification, such as subtle morphological variability, early-stage colony ambiguity, and complex multi-scale growth patterns. By improving the accuracy and stability of colony recognition, the proposed method provides more reliable technical support for microbiological analysis, clinical screening, and early fungal disease prevention. The improved detection performance highlights the potential of AI-assisted pathogen identification to enhance laboratory efficiency and reduce manual workload, thereby promoting more standardized and rapid microbial diagnostics. Looking forward, future work will focus on expanding the diversity and representativeness of biological samples, establishing larger and more complex fungal datasets, integrating clinical metadata or multi-modal information, and exploring application scenarios such as automated cultivation monitoring and intelligent clinical decision support. These efforts will further advance intelligent fungal detection toward broader applicability, higher interpretability, and greater clinical and biological value. From a practical application perspective, the real-time performance of the YOLO-CSM model makes it suitable for two key scenarios: (1) High-throughput clinical laboratory detection: The model can process a 96-well plate image in less than 0.02 seconds, enabling rapid screening of large numbers of clinical samples; (2) On-site food safety inspection: When deployed on edge devices such as NVIDIA Jetson Orin, the model can achieve real-time detection at more than 15 FPS, supporting portable detection equipment for on-site inspection of agricultural products and stored foods. These application scenarios fully demonstrate the practical value of the lightweight design and real-time performance of our model.
