# Supplementary material for: Macroscopic aspergillus recognition using YOLO-CSM
Source: PLoS One. 2026 Aug 17;21(8):e0356242. doi: 10.1371/journal.pone.0356242 (PMC13480589; doi:10.1371/journal.pone.0356242)
Supplement: Supplementary file 1 [file pone.0356242.s002.docx]

Dear Editor and Reviewers,

We sincerely appreciate your time and careful review of our manuscript, as well as the highly constructive comments and professional suggestions from the two reviewers. The reviewers’ comments have accurately identified the deficiencies and areas for improvement in the manuscript, which are of great guiding significance for enhancing the scientificity, rigor, and completeness of this study. We have organized our research team to carefully study and verify all the review comments one by one. We have conducted systematic experimental validation, data correction, and content supplementation for each issue, and comprehensively revised and polished the manuscript. The detailed revisions and responses to each comment are listed below for your review.

**Response to Reviewer 1**

**Comment 1 from the Reviewer**

The described backbone still heavily relies on convolutional layers like Conv and C3k2 alongside attention mechanisms. Why the author declare that ur YOLO improvement breaks the traditional algorithm?

**Response:**

We thank the reviewer for the valuable comments. We have carefully revised the ambiguous expressions that led to misunderstanding.

The phrase “improved and broken through traditional algorithms” in the original manuscript was imprecise. Our core innovation does **not** abandon convolutional layers (Conv/C3k2), which are essential for low-level feature extraction in object detection and cannot be replaced. We have removed the misleading statements accordingly.

The actual novelty lies in **redefining the feature extraction paradigm** of the conventional YOLO framework. Traditional YOLO variants (e.g., YOLOv11/v12) rely solely on pure CNNs for feature aggregation, which efficiently capture local details but struggle to model long-range semantic dependencies. In this study, we organically integrate the CGNet module with the C3k2 module to construct a hybrid backbone architecture for **local feature refinement and global semantic modeling**.

Specifically, the C3k2 module retains the advantages of convolutional layers in extracting local features such as edges and textures, while the CGNet module—with its dual-branch design (local feature branch and context encoding branch)—compensates for the insufficient global semantic modeling capability of traditional CNNs. This achieves deep fusion of local details and global contextual relationships.

This improvement **breaks the single pattern of “pure CNN-based feature aggregation”** in conventional YOLO, rather than restructuring the entire traditional algorithm framework. The original expression failed to clearly distinguish between “paradigm innovation” and “framework reconstruction”, causing misunderstanding. We have revised the relevant descriptions in the **Introduction** section and the paragraph introducing the YOLOv12 model in the revised manuscript to clarify the innovation and ensure academic rigor.

**Comment 2 from the Reviewer**

For vision applications in different areas, you may consider, Geometry‐Aware 3D Point Cloud Learning for Precise Cutting‐Point Detection in Unstructured Field Environments; Journal of Field Robotics. 3D vision technologies for a self-developed structural external crack damage recognition robot; Automation in Construction.

**Response:**

We thank the reviewer for recommending the high-quality academic literature. We have carefully read both papers and rigorously evaluated their relevance to our study.

The research approaches presented in the two papers provide valuable references for target feature modeling and precise localization in complex environments. However, the core task of our study is **2D macroscopic Aspergillus colony detection**, with key challenges including multi-scale variation, partial occlusion, complex background interference, and morphological irregularity of colonies in 2D images.

In contrast, the recommended literature focuses on **3D point cloud processing** and robotic detection in structured/unstructured scenes. Its technical pipeline—feature extraction and modeling based on 3D space—is fundamentally different from the 2D image analysis scenario of our work. The corresponding algorithms and model architectures are not directly transferable to our detection task.

After comprehensive evaluation, we determined that the two papers show **insufficient relevance** to our core technical route, application scenario, and key challenges. Forced citation would weaken the focus of the research narrative; therefore, we have not included them in the reference list.

We have double-checked the existing reference list to ensure all cited works are directly related to **2D fungi detection** and **YOLO model improvement**, which sufficiently support the technical innovations and conclusions of our study and meet the rigor requirements of academic citation.

**Comment 3 from the Reviewer**

The ablation study shows Recall jumps from 86.8 to 97 when combining CGNet and Shape-IoU but drops back to 87.8 in the full model.

**Response:**

We thank the reviewer for pointing out the abnormal data in the ablation experiments. After verification, this issue was caused by an error in data recording, and we have carefully corrected it as detailed below.

The Recall value (97%) for the combination “+ CGNet + Shape‑IoU” in the original Table 2 was a typo during data collation. The actual experimental result is **87%**, which has been updated in Table 2 of the revised manuscript.

This error was only a recording mistake, not a flaw in the experimental design or implementation. The corrected data show a reasonable trend consistent with the collaborative effect of the modules:

when combining CGNet and Shape‑IoU, the Recall increases steadily from 86.1% (baseline model) to 87%; after further introducing the MSCA module, the Recall is further improved to 87.8%.

This continuous improvement is consistent with our research hypothesis of “complementary enhancement by three modules”.

We have corrected the erroneous data in Table 2 and added a note explaining this revision in the **RESULTS AND DISCUSSION – Ablation Experiments** section of the revised manuscript, to ensure the accuracy and traceability of the experimental results and avoid misunderstanding caused by the recording error.

**Comment 4 from the Reviewer**

The Shape-IoU formulation uses a parameter set to 4 without justification or sensitivity analysis, making it unclear how this hyperparameter affects performance.

**Response:**

We thank the reviewer for raising the concern regarding the rigor of this parameter setting. We have supplemented the justification for choosing θ = 4, as detailed below.

θ is the nonlinear penalty coefficient of the shape cost term Ω^shape in the Shape‑IoU loss function. Its core role is to adjust the penalty intensity imposed by the model on the aspect ratio deviation of bounding boxes.

Considering the morphological characteristics of Aspergillus colonies in this study—mostly circular or elliptical, with a narrow range of aspect ratios but highly sensitive edge contours to detection accuracy—we referred to the parameter settings in the original Shape‑IoU paper [9] for similar scenarios such as microbial object localization and irregular object detection, where the reasonable range of θ is 3–5.

The choice of θ = 4 is based on two main considerations:

First, this value applies a moderate nonlinear penalty to colony shape deviation. It ensures the model accurately captures edge contours and aspect ratio features, while avoiding either localization drift caused by insufficient penalty or overfitting caused by excessive penalty.

Second, θ = 4 has been validated in the original literature as a robust choice for multi‑form, easily occluded, and small object detection, which aligns well with the core requirements of Aspergillus colony detection in this study. Thus, the rationality and reliability of this parameter setting are guaranteed without additional experiments.

We have added the above parameter justification in the **MATERIALS AND METHODS – Loss Function** section of the revised manuscript, clarifying the logical derivation and literature support for setting θ = 4, to ensure the rigor and traceability of the study.

**Comment 5 from the Reviewer**

Background removal preprocessing is mentioned but not described.

**Response:**

We thank the reviewer for pointing out this issue. We have supplemented the core background removal method in the revised manuscript:

The **Otsu adaptive thresholding algorithm** is employed. The RGB image is first converted to grayscale, and the colonies are automatically separated from the background. A mask is then generated and overlaid on the original image to retain only the colony regions, thereby eliminating background interference.

This method requires no manual parameter tuning and adapts to different experimental conditions. The relevant details have been added to the **MATERIALS AND METHODS – Dataset** section.

**Comment 6 from the Reviewer**

FPS values in Table 1 suggest YOLO-CSM is faster than YOLOv11 despite having more parameters and added modules.

**Response:**

We appreciate the reviewer’s concern regarding this issue. The main reasons are as follows:

The model size of YOLO-CSM (ModelSize = 69) is smaller than that of YOLOv11 (ModelSize = 72). The newly introduced CGNet and MSCA are lightweight modules, which do not lead to redundant parameters.

Shape-IoU only replaces the CIoU loss function in the original model. This is a **replacement-based optimization** rather than an additive extension, which does not introduce extra computational cost. Instead, it improves training and inference efficiency through more accurate shape constraints.

Therefore, the detection speed of YOLO-CSM (70.2 FPS) is higher than that of YOLOv11 (63.5 FPS).

**Comment 7 from the Reviewer**

The ethics statement says N/A but the dataset includes images collected from a hospital.

**Response:**

We thank the reviewer for pointing out this oversight. We have supplemented and refined the relevant explanation as follows:

All Aspergillus colony images used in this study were obtained from a hospital and were **artificially cultured standard strain colony images** produced by hospital laboratory technicians. They are **not clinical samples**, and do not contain any identifiable patient information or clinical diagnosis and treatment data. This study does not involve human subjects or medical privacy. Therefore, no additional ethical approval is required for this study.

The above explanation has been added to the dataset section in the revised manuscript, and the ethical statement of the paper has been fully improved accordingly.

**Comment 8 from the Reviewer**

Training used a batch size of 16 with learning rate 0.001 via Adam, u may provide discussion is provided on how these hyperparameters were tuned or validated across different model variants.

**Response:**

We thank the reviewer for raising the concern about the justification for the model training hyperparameters. We have supplemented the selection rationale and validation process for key hyperparameters including batch size, learning rate, and training epochs, as detailed below:

The hyperparameter settings in this study were determined through multiple controlled experiments and cross-validation:

A batch size of 16 was chosen based on the hardware capacity (NVIDIA RTX 4090 with 24 GB memory) and training stability. Although small-batch training converges slightly slower, it improves the precision of parameter updates. Combined with an initial learning rate of 0.001, it helps avoid gradient explosion and enhances model generalization.

An initial learning rate of 0.001 is a classic and widely adopted value for the Adam optimizer in object detection tasks. Coupled with a cosine annealing strategy, the learning rate decays dynamically, allowing the model to converge more reliably to an optimal solution in later training stages.

The training epoch of 200 was determined by monitoring the loss curves and mAP trends on both the training and validation sets. Model performance stabilizes at 200 epochs with no obvious overfitting or underfitting.

The validation process and justification for these hyperparameters have been added to the **MATERIALS AND METHODS – Experimental Environment** section of the revised manuscript to ensure the reproducibility of the study.

**Response to Reviewer 2**

**Comment 1 from the Reviewer**

Dataset description and transparency: The dataset composition is internally inconsistent (the same species appears with conflicting “rare/common” proportions). The manuscript also lacks sufficient detail to verify the dataset and prevent leakage: exact per-class sample counts, train/val/test split lists, public-source itemization and selection criteria, deduplication rules, and a clear annotation protocol (box boundary definition, occlusion/overlap handling, class-disambiguation rules). Without these, it is difficult to judge whether sample sizes are adequate and whether evaluation is unbiased.

**Response:**

We thank the reviewer for pointing out the inconsistencies and missing details in the dataset description. We have comprehensively revised and supplemented the **MATERIALS AND METHODS – Dataset** section of the paper, corrected contradictions in strain proportions, and improved key information regarding dataset validation and reproducibility. The specific revisions are as follows:

1. We corrected the typo in strain proportions. It is now clearly stated that the rare strain is *A. wentii* (15%), common strains are *A. niger* and *A. fumigatus* (20% each), and the remaining 55% consists of four other strains including *A. flavus*, ensuring logical consistency in the dataset composition.

2. We supplemented the exact sample count for each of the seven Aspergillus species, and specified that the 2174 images were split into training/validation/test sets via stratified sampling at a ratio of 8:1:1. The split list has been uploaded to the dataset repository.

3. We added filtering criteria for public data sources (resolution ≥ 1024×768, etc.) and deduplication rules (removal of images with SSIM ≥ 0.95) to standardize public data selection.

4. We established and supplemented a standardized annotation protocol, specifying rules for bounding box drawing and handling occluded/overlapped samples to ensure annotation consistency.

5. All supplementary dataset details (split list, data source entries, annotation protocol, etc.) have been uploaded to the dataset platform at https://zenodo.org/records/16412814. Only colony morphological features are retained, with no private information included.

**Comment 2,3 from the Reviewer**

2.Ablation results and internal consistency: The ablation table shows non-intuitive patterns (e.g., unusually high recall in one configuration followed by a large drop after adding an additional module; and a two-module combination outperforming the three-module combination in mAP). This conflicts with the narrative of complementary improvements. The authors should verify the table and rerun key comparisons, reporting multi-run statistics (mean±SD or confidence intervals) across multiple random seeds, along with more granular diagnostics (PR curves, per-class AP) to justify robustness.

3.Statistical rigor: The manuscript primarily reports point estimates (mAP/precision/recall) without variability measures, repeated runs, or clear controls for randomness. Given the irregular ablation behavior, the current statistical reporting is not sufficiently rigorous to support strong superiority claims.

**Response:**

We greatly appreciate the reviewer’s valuable comments. We have comprehensively revised and supplemented the manuscript regarding the consistency and statistical rigor of the ablation experiments, as detailed below:

1. We corrected data entry errors in the ablation experiment table and removed the abnormally high recall values, making the experimental results more consistent with the actual model performance.

All ablation experiments were repeated under **5 different random seeds**. We added the **mean ± standard deviation** for all performance metrics, which are uniformly presented in Table 2 of the manuscript. This strictly controls experimental randomness and improves statistical rigor.

2. We added **per-category PR curves (Fig. 5)** and **per-category AP bar charts (Fig. 6)** of the YOLO-CSM model in the visualization analysis section, verifying the detection robustness of the model for different abundance Aspergillus strains at a fine-grained level.

3. Based on the supplemented statistical results and newly added figures, we refined the analysis and description of the experimental results in the manuscript, further demonstrating the complementary optimization effect of each module, and strengthening the evidence supporting the research conclusions.

**Comment 4 from the Reviewer**

Presentation and labeling: Some figures are too low-resolution to enable replication (e.g., architecture diagrams). Label naming is inconsistent between visualizations and the species names used in text (e.g., non-standard labels appearing in detection examples), which prevents readers from mapping outputs to classes. The label taxonomy should be unified across annotation, text, and figures.

**Response:**

We greatly appreciate the reviewer for pointing out the issues regarding figure presentation and labeling. These comments are highly valuable for improving the readability, standardization, and reproducibility of the manuscript. We have comprehensively revised and optimized the relevant issues as follows:

Improve the quality of low-resolution figures:

Figures with insufficient resolution (such as the model architecture diagram) have been redrawn and exported in high-resolution formats to ensure clear and legible details, meeting the visual requirements for research reproduction.

Unify the labeling and classification system throughout the manuscript:

We have thoroughly checked and unified the classification system for strain names and figure labels. Non-standard labels in visualization results (e.g., detection examples) have been revised to ensure **full consistency** of Aspergillus strain names and category labels across dataset annotations, main text, and all figures (including detection results, PR curves, AP bar charts, etc.), with unambiguous correspondence.
