# Supplementary material for: Macroscopic aspergillus recognition using YOLO-CSM
Source: PLoS One. 2026 Aug 17;21(8):e0356242. doi: 10.1371/journal.pone.0356242 (PMC13480589; doi:10.1371/journal.pone.0356242)
Supplement: Supplementary file 2 [file pone.0356242.s003.docx]

Dear Reviewer #2,

We sincerely appreciate your rigorous review and valuable comments on our manuscript. Your insightful suggestions are of great importance for improving the scientific rigor and data accuracy of our study. We have carefully addressed the two issues you raised, conducted a full and systematic verification of all data in the manuscript, and completed corresponding revisions and corrections. The detailed responses to your comments are as follows:

**Comment 1 from the Reviewer**

There is still an obvious mathematical inconsistency in the description of the dataset composition. The manuscript states that the rare strain accounts for 15%, two common strains account for 20% each, and the remaining four strains account for 55%. These proportions sum to 110%, which is mathematically impossible. This is also inconsistent with the authors’ claim that the dataset description has been corrected and made logically consistent. I recommend that the authors carefully recheck this part and report the exact sample number and proportion for each category directly in the manuscript.

**Response:**

We apologize for the careless numerical error in the proportion description of the dataset in the previous revised version, which led to the illogical sum of proportions. We have rechecked and revised the dataset part in detail: the exact sample number and actual accounting proportion of each of the seven Aspergillus species are now clearly reported directly in the manuscript, and the proportion of each category is calculated strictly based on the total sample size of 2174 images, with the sum of all proportions being 100%, completely resolving the previous mathematical inconsistency. The revised specific content is as follows:

The rare strain *Aspergillus wentii* consists of 219 images (approximately 10%); the common strains *Aspergillus niger* and *Aspergillus fumigatus* comprise 498 and 408 images, accounting for approximately 23% and 19% respectively; the remaining strains include *Aspergillus flavus* (188 images), *Aspergillus versicolor* (170 images), *Aspergillus terreus* (325 images) and *Aspergillus candidus* (366 images). The exact sample size and proportion of each strain are clearly presented to ensure the logical consistency of dataset description.

**Comment 2 from the Reviewer**

The reliability of numerical checking in the manuscript still appears insufficient. The authors have already acknowledged a previous recording error in the ablation table, where an abnormal Recall value was reported incorrectly. Together with the dataset proportion error that still remains in the revised manuscript, this suggests that the numerical values in the text, tables, and corresponding descriptions may not have been fully and systematically verified. I recommend that the authors conduct a thorough check of all reported data, proportions, and performance metrics to ensure consistency and accuracy throughout the manuscript.

**Response:**

We fully agree with your comment that the numerical checking reliability needs to be improved. Aiming at the previous recording error of the Recall value in the ablation table and the dataset proportion error, we have organized the research team to conduct a full, systematic and thorough verification of all numerical data in the manuscript, including all performance metrics in the text, tables and figure annotations (Precision, Recall, mAP, FPS, etc.), dataset sample sizes and proportions, experimental parameter settings, and other key numerical information. We have carefully checked the consistency between the numerical values in the text description, tables and figure labels, corrected all careless errors found in the verification process, and ensured that all reported data, proportions and performance metrics are accurate and consistent throughout the manuscript.
