# Supplementary material for: Macroscopic aspergillus recognition using YOLO-CSM
Source: PLoS One. 2026 Aug 17;21(8):e0356242. doi: 10.1371/journal.pone.0356242 (PMC13480589; doi:10.1371/journal.pone.0356242)
Supplement: Supplementary file 3 [file pone.0356242.s004.docx]

Dear Editor and Reviewers,

We sincerely appreciate your time and careful review of our manuscript, as well as the highly constructive comments and professional suggestions from the two reviewers. The reviewers’ comments have accurately identified the deficiencies and areas for improvement in the manuscript, which are of great guiding significance for enhancing the scientificity, rigor, and completeness of this study. We have organized our research team to carefully study and verify all the review comments one by one. We have conducted systematic experimental validation, data correction, and content supplementation for each issue, and comprehensively revised and polished the manuscript. The detailed revisions and responses to each comment are listed below for your review.

**Response to Reviewer 3**

**Comment 1 from the Reviewer**

The fundamental motivation of the study is insufficiently established. The authors broadly discuss the importance of fungal detection, but they do not adequately justify why object detection is the optimal approach for this specific task compared to image classification or instance segmentation. Furthermore, the rationale for selecting the recently released YOLOv12 as the baseline is unclear.

**Response:**

We sincerely appreciate your constructive comment. We have revised the Introduction to clarify:

1.Object detection is more suitable than classification or segmentation, as it can simultaneously identify, locate and count multiple colonies in one image, which fits real laboratory needs.

2.YOLOv12 is selected as the baseline because we compared several advanced models, and YOLOv12 showed more stable and balanced performance on our Aspergillus dataset.

All revisions have been marked in the manuscript.

**Comment 2 from the Reviewer**

In the comparative experiments with state-of-the-art (SOTA) models, the authors only report Precision, mAP, Model Size, and FPS, notably omitting Recall. The authors must explain why Recall was excluded from this SOTA comparison and discuss its significance, especially when detecting early-stage or microscopic fungal strains.

**Response:**

We sincerely appreciate your constructive comment. Recall was not omitted intentionally. In our revised manuscript, we have added Recall into Table 1 to make the comparison with SOTA models more comprehensive. Recall is particularly critical for detecting early-stage and weak-feature fungal strains, as it reflects the model’s ability to reduce missed detections. All relevant metrics are now fully reported and consistent.

**Comment 3 from the Reviewer**

The dataset contains 2,174 images distributed across seven Aspergillus species, averaging roughly 300 images per class. With an 8:1:1 split for training, validation, and testing, the test set only contains around 217 images. This sample size is too small to statistically guarantee robust evaluation across seven classes. The authors must investigate and clarify whether training on such limited data leads to overfitting.

**Response:**

We sincerely appreciate your constructive comment. During training, we closely monitored the loss and evaluation metrics of both the training and validation sets. The results showed stable convergence without a significant gap between training and validation performance, indicating no obvious overfitting risk in our experiments.

**Comment 4 from the Reviewer**

Removing the background explicitly contradicts the authors' later claims that the MSCA module improves robustness to "complex backgrounds".

**Response:**

We sincerely appreciate your careful comment. Background removal only eliminates simple and uniform interference in images. MSCA is proposed to enhance robustness against complex background disturbances such as marker marks, tape, and occlusions, which are not fully removed by preprocessing. This is consistent with our description.

**Comment 5 from the Reviewer**

Given this powerful hardware (NVIDIA GeForce RTX 4090 GPU), the practical significance of introducing the lightweight CGNet module to reduce computational costs is not convincingly demonstrated.

**Response:**

We sincerely appreciate your constructive comment. The proposed lightweight CGNet module is not designed for high‑end GPUs such as the RTX 4090. **We have also conducted experiments on low‑end and low‑power GPUs**, and the results confirm that the lightweight design effectively reduces computational load and improves inference speed in resource‑limited environments. This demonstrates the practical value and deployment potential of the proposed module.

**Comment 6 from the Reviewer**

While Shape-IoU is introduced to optimize localization for targets with complex morphologies and occlusions, the visual detection results primarily display relatively regular and well-defined colonies, failing to highlight the specific value of this loss function.

**Response:**

We sincerely appreciate your constructive comment. Shape‑IoU is proposed to improve localization for occluded, blurred, and irregular colonies. In our visualization results, we have presented detection cases with marker pen occlusion and complex interference, which can fully reflect the practical value and effectiveness of the Shape‑IoU loss function.

**Comment 7 from the Reviewer**

The benefits of the MSCA module in handling complex scenarios are not evident in the provided case studies.

**Response:**

We sincerely appreciate your constructive comment. The MSCA module is designed to enhance detection in complex scenarios such as occlusion, overlapping colonies, marker interference, and uneven illumination. Our visualized case studies have included typical challenging samples with these disturbances, and the ablation results further confirm that MSCA effectively improves detection stability and accuracy in complex backgrounds.

**Comment 8 from the Reviewer**

The ablation study further reveals that the performance gains of YOLO-CSM over the baseline YOLOv12 are marginal (e.g., mAP increases from 87.2% to 89.2%, and Recall only increases from 86.1% to 87.8%).

**Response:**

We sincerely appreciate your constructive comment. Although the absolute improvement of mAP and Recall appears numerically modest, **the enhancement is statistically consistent and practically meaningful** for fungal colony detection, especially in reducing missed detections and improving localization accuracy for occluded, small, and irregular strains. Furthermore, combined with the lightweight design and robust performance in complex backgrounds, the overall improvement of the model is comprehensive and valuable for real-world application.

**Comment 9 from the Reviewer**

To truly demonstrate the proposed model's value, the authors should test it on complex, real-world scenarios with blurred boundaries, heavy occlusions, and without artificial background removal.

**Response:**

We sincerely appreciate your constructive comment. The artificial background removal in our preprocessing only eliminates simple, uniform, and irrelevant background regions (e.g., pure culture dish areas). All experiments were inherently conducted under realistic complex backgrounds that still contain marker marks, tape traces, occlusions, uneven illumination, and overlapping colonies, which are the key challenging factors we focused on. Therefore, the evaluation fully reflects the model’s performance in real-world complex scenarios.

**Comment 10 from the Reviewer**

The literature review is severely limited, containing only 14 references. The authors must significantly expand the scope and depth of their literature review to adequately contextualize their contributions within the broader fields of deep learning-based object detection, attention mechanisms, and automated mycology.

**Response:**

We sincerely appreciate your constructive comment. We have significantly expanded the literature review by supplementing a large number of representative and latest references related to deep learning object detection, attention mechanism design and intelligent fungal detection. The revised introduction fully combs the research progress in related fields, and clearly positions the innovation and contribution of this paper in the existing research system

**Response to Reviewer 4**

**Comment 1 from the Reviewer**

The improved model structure diagram of YOLO-CSM is missing.

**Response:**

We sincerely appreciate your constructive comment. We have supplemented the complete structure diagram of the improved YOLO-CSM model in the manuscript, which clearly shows the overall network architecture and the integration of each proposed module, making the model design more intuitive and comprehensive.

**Comment 2 from the Reviewer**

The model structure diagrams of the key modules CGNet and MSCA are missing.

**Response:**

We sincerely appreciate your constructive comment. We have added the detailed model structure diagrams of the key modules, including CGNet and MSCA, in the manuscript to clearly present their internal design and working mechanism, so as to make the module structure more intuitive and complete.

**Comment 3 from the Reviewer**

The schematic diagram illustrating the principle of Shape-IoU is missing.

**Response:**

We sincerely appreciate your constructive comment. We have supplemented the schematic diagram illustrating the working principle of Shape-IoU in the manuscript to clearly show its mechanism for optimizing bounding box regression, making the design of the loss function more intuitive and comprehensive.

**Comment 4 from the Reviewer**

In the Ablation Experiments section, the term “random seeds” in the sentence “All experiments were repeated five times with different random seeds to ensure the reliability of results” is not clearly explained. Given that the dataset split is fixed at 8:1:1, the statement “repeating experiments five times with different random seeds” is likely to cause confusion for readers.

**Response:**

We sincerely appreciate your careful comment. The random seeds are only used to initialize the model training process, not for dataset partitioning. The dataset split is fixed at 8:1:1 in all experiments, and different random seeds are merely employed to reduce the randomness of network training and ensure the stability and repeatability of the experimental results.

**Comment 5 from the Reviewer**

Approximately 15% of the images in the dataset contain partial occlusions (e.g., tape, marker traces). During preprocessing, only colonies with an occlusion rate < 50% were labeled “occluded”. However, the manuscript does not elaborate on the specific calculation method of the occlusion rate, nor does it clarify whether samples with the occlusion label are specially treated in training and evaluation (such as through data augmentation or weighted loss functions). This reduces the transparency of the argument regarding the method’s adaptability to occlusion scenarios.

**Response:**

We sincerely appreciate your comment. The occlusion rate refers to the ratio of the occluded area to the colony bounding box area. We used data augmentation for occluded samples, and applied Shape-IoU and MSCA to improve occlusion robustness, without extra weighted loss.

**Comment 6 from the Reviewer**

A comparison of Recall (R) is missing in Table 1. In object detection tasks, Precision (P) and Recall (R) are a pair of core and mutually constraining metrics that jointly reflect the comprehensive detection performance of a model (usually summarized by mAP). Since Table 1 only provides P and mAP, readers cannot assess how YOLO-CSM compares with other models in terms of recall. For example, although YOLO-CSM achieves the highest precision, it remains unclear whether its recall is also superior to or at least comparable with that of YOLOv11. Without the R values, the comparison is incomplete.

**Response:**

We sincerely appreciate your constructive comment. We have added a Recall (R) column to Table 1 to make the model comparison complete and comprehensive. The results clearly show that YOLO-CSM achieves the highest Recall (87.8%) among all compared methods, which is notably better than YOLOv11 and other baseline models. This fully verifies its superior and balanced overall detection performance.

**Comment 7 from the Reviewer**

The purpose of the “occluded” label is unclear. The manuscript only mentions that this label was assigned, but does not state at all whether the label serves any function in subsequent processes.

**Response:**

We sincerely appreciate your constructive comment. The occluded label is only used during dataset annotation and statistical description to mark and count occluded samples. It does not participate in model training, loss calculation, or the evaluation process, and has no impact on the training and testing of the model.
