# Supplementary material for: Macroscopic aspergillus recognition using YOLO-CSM
Source: PLoS One. 2026 Aug 17;21(8):e0356242. doi: 10.1371/journal.pone.0356242 (PMC13480589; doi:10.1371/journal.pone.0356242)
Supplement: Supplementary file 4 [file pone.0356242.s005.docx]

Dear Editor and Reviewers,

We sincerely appreciate your time and careful review of our manuscript, as well as the highly constructive comments and professional suggestions from the three reviewers. The reviewers’ comments have accurately identified the deficiencies and areas for improvement in the manuscript, which are of great guiding significance for enhancing the scientificity, rigor, and completeness of this study. We have organized our research team to carefully study and verify all the review comments one by one. We have conducted systematic experimental validation, data correction, and content supplementation for each issue, and comprehensively revised and polished the manuscript. The detailed revisions and responses to each comment are listed below for your review.

**Response to Reviewer 4**

**Comment 1 from the Reviewer**

The paper does not provide the figures for "Fig. 1 Schematic diagram of partial data from Aspergillus dataset" and "Fig. 2 Schematic Diagram of the YOLO12 Model Architecture."

**Response:**

Thank you for the valuable comment.We have carefully checked the manuscript and found that the original figures were not properly displayed in the submitted version.Fig.1 (Dataset Samples) and Fig.2 (YOLOv12 Architecture) have now been reinserted and uploaded in high resolution in the revised manuscript.The corresponding figure captions have also been checked and updated.

**Comment 2 from the Reviewer**

"Fig. 3 The improved YOLO-CSM architecture" is too vague to see clearly.

**Response:**

We agree with the reviewer.The original Fig.3 suffered from insufficient resolution.We have redrawn the YOLO-CSM architecture and replaced it with a high-resolution version. The structure of CGNet, Shape-IoU and MSCA integration is now clearly visible.

**Comment 3 from the Reviewer**

From "Fig. 4 The structure of CGNet," it is impossible to discern how the authors integrated CGNet into the YOLOv12 model; therefore, an in-depth critique of the paper cannot be provided.

**Response:**

Thank you for your valuable comment and for pointing out this critical ambiguity in our manuscript. We sincerely apologize for the insufficient clarity in the previous figure design and text description, which made it difficult to understand the integration of CGNet into the YOLOv12 framework. We have comprehensively revised the relevant sections and figures to address this issue thoroughly.

Specifically, the following modifications have been made in the revised manuscript:

1.Revised Figure 3 (YOLO-CSM architecture): We have enhanced the annotations in Figure 3 to explicitly mark the two C3k2-CGNet blocks (highlighted in red) that replace the original C3k2 modules in the backbone network. Clear labels indicating "Stage 2" and "Stage 3" have been added to the backbone section, and arrows have been inserted to show the feature flow through the modified modules.

2.Supplemented detailed text description in Section 3.2: We have added the following explicit statement immediately after introducing the CGNet module: "Specifically, the original C3k2 module in the backbone was replaced by the proposed C3k2-CGNet block. The modified blocks are located in Stage 2 and Stage 3 of the backbone network." This directly clarifies the exact integration position and method.

3.Clarified the division of labor between Figure 3 and Figure 4: We have revised the figure legends to explicitly state that Figure 3 illustrates the overall architecture of the improved YOLO-CSM model and shows where CGNet is integrated into the YOLOv12 backbone, while Figure 4 only presents the internal structure of the standalone CGNet module.

These revisions clearly demonstrate that we replaced the original C3k2 modules in Stage 2 and Stage 3 of the YOLOv12 backbone with our proposed C3k2-CGNet blocks, rather than inserting CGNet as a separate standalone module. We believe these comprehensive modifications have fully resolved the ambiguity you raised. If you require any further clarification or additional annotations, please do not hesitate to let us know.

**Comment 4 from the Reviewer**

The response to "Comment 4 from the Reviewer" is unsatisfactory. The author replied, "different random seeds are merely employed to reduce the randomness of network training and ensure the stability and repeatability of the experimental results." However, the revised manuscript does not provide a detailed explanation of howthis was specifically done.

**Response:**

Thank you for your valuable comment and for pointing out this deficiency. We apologize for the insufficient explanation in the previous response and have now supplemented the detailed implementation of the random seed strategy in the revised manuscript.

Specifically, our random seed control and experimental repeatability guarantee measures are implemented as follows:

1.Independent repeated experiments: All ablation experiments and comparative experiments were independently repeated 5 times using different random seeds.

2.Standardized seed selection: We adopted 5 widely recognized integer random seeds (123, 456, 789, 101112, 131415) in the computer vision field to ensure the universality and reproducibility of the experimental results.

3.Full random source fixation: To completely eliminate other potential random interference factors, we fixed all random sources during the entire training process, including the random seed for training set shuffling, the initialization seed of model weights, and the random seed for all data augmentation operations (such as random flipping, random cropping, and color jitter).

4.Statistical presentation of results: For each experimental configuration, we calculated the mean value and standard deviation of the core detection metrics (Precision, Recall, mAP) across the 5 independent runs, and presented the results in the form of "mean±standard deviation" in Table 2 (Ablation Experiments) of the manuscript. This statistical method can objectively reflect the stability and fluctuation range of the model performance.

The above detailed description has been added to the first paragraph of the "Ablation Experiments" section (Section 4.2) in the revised manuscript. We believe that this comprehensive explanation can fully address your concern about the experimental repeatability. If you have any further questions, please do not hesitate to let us know.

**Response to Reviewer 5**

Thank you very much for your positive and encouraging comments on our revised manuscript. We greatly appreciate your careful review and affirmation of our improvements to the paper. We will cooperate with the journal to complete subsequent minor editorial revisions as required.

**Response to Reviewer 6**

**Comment 1 from the Reviewer**

The introduction only includes eight references. Is this sufficient to support the focused problem definition, research gaps, and contributions of this paper?

**Response:**

Thank you for your valuable comment. We acknowledge that the original 8 references were insufficient to fully support our work. We have thoroughly revised the introduction and added 11 high-quality peer-reviewed references, all organically integrated into its logical structure. The revised introduction now provides a solid foundation for our problem definition, research gaps and contributions. We welcome any further suggestions for relevant literature.

**Comment 2 from the Reviewer**

The introduction mentions real-time performance of the proposed framework, but this is not demonstrated in subsequent application scenarios.

**Response:**

Thank you for pointing out this important issue. We acknowledge that the real-time performance of our framework was not sufficiently demonstrated in the application scenarios section. We have now added detailed inference speed analysis and real-world deployment feasibility discussion in the revised manuscript. Specifically, we supplemented the FPS comparison results with mainstream models in Table 1, and explained how the 70.2 FPS inference speed meets the requirements of high-throughput clinical laboratory detection and on-site food safety inspection scenarios. These additions fully demonstrate the practical value of our model's real-time performance.

**Comment 3 from the Reviewer**

In the Experimental Environment section, the authors state that "Based on multiple rounds of experiments and cross-validation, the batch size was set to 16." Please provide supplementary experimental results.

**Response:**

Thank you for your comment. The selection of batch size = 16 was finalized via repeated five-fold cross-validation during our preliminary debugging phase with multiple repeated training runs. Due to limited experimental equipment and dataset partitioning rules fixed in our original experimental scheme, additional supplementary ablation tests for other batch sizes cannot be newly conducted. However, we have added a detailed textual description of the cross-validation process and parameter selection basis in the revised manuscript to explain why we chose 16 as the final batch size.

**Comment 4 from the Reviewer**

The authors cite the CGNet module, a method proposed in 2019. Many lightweight modules are available today. Why did the authors choose this one? Please provide a detailed explanation of its principles and a comparison with other lightweight modules.

**Response:**

Thank you for your constructive comment. We have supplemented the principle of CGNet and comparative analysis with mainstream lightweight modules (MobileNetV3, ShuffleNetV2, GhostModule) in the revised manuscript.

We select CGNet mainly for task adaptation: our research targets tiny early Aspergillus colonies with blurry features. Different from conventional lightweight modules that only cut parameters, CGNet introduces dual-branch context-guided convolution to capture local details and global contextual information simultaneously at low computational cost, significantly improving small-colony detection. MobileNetV3, ShuffleNetV2 and GhostModule pursue extreme lightweight but lack long-range context modeling, causing feature loss for small fungal targets. Therefore, CGNet achieves the best balance between lightweight computation and detection accuracy for our dataset.

**Comment 5 from the Reviewer**

In the comparative experiments in RESULTS AND DISCUSSION, please add a comparison between the latest YOLOv13 and YOLOv26. Appropriate deep learning modeling techniques are essential to ensure the quality of the constructed model. For this reason, some cutting-edge research works have emerged. For example, Growth monitoring of rice based on UAV hyperspectral images and improved deep learning method: The QRCNN-BIGRU-MLLA network, ImobileTransformer: a fusion-based lightweight model for rice disease identification. If possible, related work needs to be mentioned.

**Response:**

Thank you for your valuable suggestions. The whole experimental work of this manuscript was finished prior to the release of YOLOv13 and YOLOv26. Retroactive testing cannot guarantee identical experimental settings and fair comparison, so their comparative data cannot be supplemented in this revision. We have supplemented YOLOv12 with complete official open resources for latest-model comparison.

**Comment 6 from the Reviewer**

In the RESULTS AND DISCUSSION section, it is recommended to add a visual comparison of the detection results of different models on the same image.

**Response:**

Thank you very much for your kind and valuable suggestion.

We fully agree that visual comparison of detection results can intuitively reflect model performance, and cross-model comparison is also helpful to observe the performance differences among various algorithms. All detection illustrations already presented in this manuscript are representative test results from our optimal YOLO-CSM model.

**Comment 7 from the Reviewer**

The MSCA module itself is not an original work of the authors; improvements or adaptations to the MSCA module in this paper should be clearly indicated.

**Response:**

Thank you for your careful comment.

The MSCA module is adopted from existing published work, and we have cited the corresponding reference in the manuscript.
