# Supplementary material for: Macroscopic aspergillus recognition using YOLO-CSM
Source: PLoS One. 2026 Aug 17;21(8):e0356242. doi: 10.1371/journal.pone.0356242 (PMC13480589; doi:10.1371/journal.pone.0356242)
Supplement: Peer review history

# Peer review: Decision Letter 0

**DOI:** 10.1371/journal.pone.0356242.r001

**Authors:** Gayathiri Ekambaram

9 Feb 2026

Dear Dr. Ru,

Thank you for submitting your manuscript to PLOS ONE. After careful consideration, we feel that it has merit but does not fully meet PLOS ONE’s publication criteria as it currently stands. Therefore, we invite you to submit a revised version of the manuscript that addresses the points raised during the review process.

Please submit your revised manuscript by Mar 26 2026 11:59PM. If you will need more time than this to complete your revisions, please reply to this message or contact the journal office at plosone@plos.org. When you're ready to submit your revision, log on to https://www.editorialmanager.com/pone/ and select the 'Submissions Needing Revision' folder to locate your manuscript file.

- A letter that responds to each point raised by the academic editor and reviewer(s). You should upload this letter as a separate file labeled 'Response to Reviewers'.
- A marked-up copy of your manuscript that highlights changes made to the original version. You should upload this as a separate file labeled 'Revised Manuscript with Track Changes'.
- An unmarked version of your revised paper without tracked changes. You should upload this as a separate file labeled 'Manuscript'.

If you would like to make changes to your financial disclosure, please include your updated statement in your cover letter. Guidelines for resubmitting your figure files are available below the reviewer comments at the end of this letter.

If applicable, we recommend that you deposit your laboratory protocols in protocols.io to enhance the reproducibility of your results. Protocols.io assigns your protocol its own identifier (DOI) so that it can be cited independently in the future. For instructions see: https://journals.plos.org/plosone/s/submission-guidelines#loc-laboratory-protocols. Additionally, PLOS ONE offers an option for publishing peer-reviewed Lab Protocol articles, which describe protocols hosted on protocols.io. Read more information on sharing protocols at https://plos.org/protocols?utm_medium=editorial-email&utm_source=authorletters&utm_campaign=protocols.

We look forward to receiving your revised manuscript.

Kind regards,

Gayathiri Ekambaram, Ph.D

Academic Editor

PLOS One

Journal requirements:

When submitting your revision, we need you to address these additional requirements.

1. Please ensure that your manuscript meets PLOS ONE's style requirements, including those for file naming. The PLOS ONE style templates can be found at

https://journals.plos.org/plosone/s/file?id=wjVg/PLOSOne_formatting_sample_main_body.pdf and

https://journals.plos.org/plosone/s/file?id=ba62/PLOSOne_formatting_sample_title_authors_affiliations.pdf

2. Please note that PLOS One has specific guidelines on code sharing for submissions in which author-generated code underpins the findings in the manuscript. In these cases, we expect all author-generated code to be made available without restrictions upon publication of the work. Please review our guidelines at https://journals.plos.org/plosone/s/materials-and-software-sharing#loc-sharing-code and ensure that your code is shared in a way that follows best practice and facilitates reproducibility and reuse.

3. Thank you for stating the following financial disclosure:

“This work was supported by the Three Three Three Talent Project of Hebei Province in 2023 (No. C20231019), the Key Team Project of Basic Scientific Research Business Expense Program of Hebei University of Architecture (No. 2025ZDTD04), the 2025 Hebei Provincial Innovation Capability Enhancement Program – Soft Science Research Special Project (No. 25357635D), and the 2026 Sports Science and Technology Research Projects of Hebei Provincial Sports Bureau (No. 2026CY44). The authors gratefully acknowledge this support.”

Please state what role the funders took in the study.  If the funders had no role, please state: "The funders had no role in study design, data collection and analysis, decision to publish, or preparation of the manuscript."

If this statement is not correct you must amend it as needed.

Please include this amended Role of Funder statement in your cover letter; we will change the online submission form on your behalf.

4. When completing the data availability statement of the submission form, you indicated that you will make your data available on acceptance. We strongly recommend all authors decide on a data sharing plan before acceptance, as the process can be lengthy and hold up publication timelines. Please note that, though access restrictions are acceptable now, your entire data will need to be made freely accessible if your manuscript is accepted for publication. This policy applies to all data except where public deposition would breach compliance with the protocol approved by your research ethics board. If you are unable to adhere to our open data policy, please kindly revise your statement to explain your reasoning and we will seek the editor's input on an exemption. Please be assured that, once you have provided your new statement, the assessment of your exemption will not hold up the peer review process.

5. PLOS requires an ORCID iD for the corresponding author in Editorial Manager on papers submitted after December 6th, 2016. Please ensure that you have an ORCID iD and that it is validated in Editorial Manager. To do this, go to ‘Update my Information’ (in the upper left-hand corner of the main menu), and click on the Fetch/Validate link next to the ORCID field. This will take you to the ORCID site and allow you to create a new iD or authenticate a pre-existing iD in Editorial Manager.

If the reviewer comments include a recommendation to cite specific previously published works, please review and evaluate these publications to determine whether they are relevant and should be cited. There is no requirement to cite these works unless the editor has indicated otherwise.

Additional Editor Comments:

Dear Wenjie Zhao and colleagues,

The reviewers find your work on the macroscopic detection of Aspergillus colonies through an improved YOLOv12 framework to be a relevant and technically interesting application of deep learning in microbiology. However, both reviewers have raised significant concerns regarding the internal consistency of your data, the statistical rigor of your results, and potential errors in your ablation study reporting.

Based on these evaluations, I am requesting a Major Revision. Your revised manuscript must address the critical points asked by the Reviewers

[Note: HTML markup is below. Please do not edit.]

Reviewers' comments:

Reviewer's Responses to Questions

**Comments to the Author**

1. Is the manuscript technically sound, and do the data support the conclusions?

Reviewer #1: Yes

Reviewer #2: Partly

**********

2. Has the statistical analysis been performed appropriately and rigorously?

Reviewer #1: Yes

Reviewer #2: No

**********

3. Have the authors made all data underlying the findings in their manuscript fully available?

The PLOS Data policy

Reviewer #1: Yes

Reviewer #2: Yes

**********

4. Is the manuscript presented in an intelligible fashion and written in standard English?

Reviewer #1: Yes

Reviewer #2: Yes

**********

Reviewer #1: I recommend minor revision.

The authors propose a modified YOLOv12 architecture integrating CGNet, Shape-IoU, and MSCA modules for macroscopic Aspergillus colony detection, aiming to improve performance under occlusion, multi-scale variation, and complex backgrounds. The manuscript reports promising metrics on a custom dataset and includes ablation studies.

(1)The described backbone still heavily relies on convolutional layers like Conv and C3k2 alongside attention mechanisms. Why the author declare that ur YOLO improvement breaks the traditional algorithm?

(2)For vision applications in different areas, you may consider, Geometry‐Aware 3D Point Cloud Learning for Precise Cutting‐Point Detection in Unstructured Field Environments; Journal of Field Robotics. 3D vision technologies for a self-developed structural external crack damage recognition robot; Automation in Construction.

(3)The ablation study shows Recall jumps from 86.8 to 97 when combining CGNet and Shape-IoU but drops back to 87.8 in the full model.

(4)The Shape-IoU formulation uses a parameter set to 4 without justification or sensitivity analysis, making it unclear how this hyperparameter affects performance.

(5)Background removal preprocessing is mentioned but not describe.

(6)FPS values in Table 1 suggest YOLO-CSM is faster than YOLOv11 despite having more parameters and added modules.

(7)The ethics statement says N/A but the dataset includes images collected from a hospital.

(8)Training used a batch size of 16 with learning rate 0.001 via Adam, u may provide discussion is provided on how these hyperparameters were tuned or validated across different model variants.

Reviewer #2: The study addresses a relevant problem (macroscopic detection of Aspergillus colonies) and the overall approach—building on a standard detection framework and evaluating with common metrics—is technically feasible. However, in its current form the evidence supports the conclusions only partly, due to major issues in dataset reporting, reproducibility, and result consistency.

1.Dataset description and transparency: The dataset composition is internally inconsistent (the same species appears with conflicting “rare/common” proportions). The manuscript also lacks sufficient detail to verify the dataset and prevent leakage: exact per-class sample counts, train/val/test split lists, public-source itemization and selection criteria, deduplication rules, and a clear annotation protocol (box boundary definition, occlusion/overlap handling, class-disambiguation rules). Without these, it is difficult to judge whether sample sizes are adequate and whether evaluation is unbiased.

2.Ablation results and internal consistency: The ablation table shows non-intuitive patterns (e.g., unusually high recall in one configuration followed by a large drop after adding an additional module; and a two-module combination outperforming the three-module combination in mAP). This conflicts with the narrative of complementary improvements. The authors should verify the table and rerun key comparisons, reporting multi-run statistics (mean±SD or confidence intervals) across multiple random seeds, along with more granular diagnostics (PR curves, per-class AP) to justify robustness.

3.Statistical rigor: The manuscript primarily reports point estimates (mAP/precision/recall) without variability measures, repeated runs, or clear controls for randomness. Given the irregular ablation behavior, the current statistical reporting is not sufficiently rigorous to support strong superiority claims.

4.Presentation and labeling: Some figures are too low-resolution to enable replication (e.g., architecture diagrams). Label naming is inconsistent between visualizations and the species names used in text (e.g., non-standard labels appearing in detection examples), which prevents readers from mapping outputs to classes. The label taxonomy should be unified across annotation, text, and figures.

Overall, the technical direction is plausible, but substantial corrections and stronger reproducibility/statistical reporting are required before the main conclusions can be considered well supported.

**********

what does this mean?). If published, this will include your full peer review and any attached files.

If you choose “no”, your identity will remain anonymous but your review may still be made public.

**Do you want your identity to be public for this peer review?**  For information about this choice, including consent withdrawal, please see our Privacy Policy

Reviewer #1: No

Reviewer #2: No

**********

[NOTE: If reviewer comments were submitted as an attachment file, they will be attached to this email and accessible via the submission site. Please log into your account, locate the manuscript record, and check for the action link "View Attachments". If this link does not appear, there are no attachment files.]

To ensure your figures meet our technical requirements, please review our figure guidelines: https://journals.plos.org/plosone/s/figures

You may also use PLOS’s free figure tool, NAAS, to help you prepare publication quality figures: https://journals.plos.org/plosone/s/figures#loc-tools-for-figure-preparation.

NAAS will assess whether your figures meet our technical requirements by comparing each figure against our figure specifications.

# Author comment: Author response to Decision Letter 1

**DOI:** 10.1371/journal.pone.0356242.r002

25 Feb 2026

Dear Editor and Reviewers,

We sincerely appreciate your time and careful review of our manuscript, as well as the highly constructive comments and professional suggestions from the two reviewers. The reviewers’ comments have accurately identified the deficiencies and areas for improvement in the manuscript, which are of great guiding significance for enhancing the scientificity, rigor, and completeness of this study. We have organized our research team to carefully study and verify all the review comments one by one. We have conducted systematic experimental validation, data correction, and content supplementation for each issue, and comprehensively revised and polished the manuscript. The detailed revisions and responses to each comment are listed below for your review.

Response to Reviewer 1

Comment 1 from the Reviewer

The described backbone still heavily relies on convolutional layers like Conv and C3k2 alongside attention mechanisms. Why the author declare that ur YOLO improvement breaks the traditional algorithm?

Response:

We thank the reviewer for the valuable comments. We have carefully revised the ambiguous expressions that led to misunderstanding.

The phrase “improved and broken through traditional algorithms” in the original manuscript was imprecise. Our core innovation does not abandon convolutional layers (Conv/C3k2), which are essential for low-level feature extraction in object detection and cannot be replaced. We have removed the misleading statements accordingly.

The actual novelty lies in redefining the feature extraction paradigm of the conventional YOLO framework. Traditional YOLO variants (e.g., YOLOv11/v12) rely solely on pure CNNs for feature aggregation, which efficiently capture local details but struggle to model long-range semantic dependencies. In this study, we organically integrate the CGNet module with the C3k2 module to construct a hybrid backbone architecture for local feature refinement and global semantic modeling.

Specifically, the C3k2 module retains the advantages of convolutional layers in extracting local features such as edges and textures, while the CGNet module—with its dual-branch design (local feature branch and context encoding branch)—compensates for the insufficient global semantic modeling capability of traditional CNNs. This achieves deep fusion of local details and global contextual relationships.

This improvement breaks the single pattern of “pure CNN-based feature aggregation” in conventional YOLO, rather than restructuring the entire traditional algorithm framework. The original expression failed to clearly distinguish between “paradigm innovation” and “framework reconstruction”, causing misunderstanding. We have revised the relevant descriptions in the Introduction section and the paragraph introducing the YOLOv12 model in the revised manuscript to clarify the innovation and ensure academic rigor.

Comment 2 from the Reviewer

For vision applications in different areas, you may consider, Geometry‐Aware 3D Point Cloud Learning for Precise Cutting‐Point Detection in Unstructured Field Environments; Journal of Field Robotics. 3D vision technologies for a self-developed structural external crack damage recognition robot; Automation in Construction.

Response:

We thank the reviewer for recommending the high-quality academic literature. We have carefully read both papers and rigorously evaluated their relevance to our study.

The research approaches presented in the two papers provide valuable references for target feature modeling and precise localization in complex environments. However, the core task of our study is 2D macroscopic Aspergillus colony detection, with key challenges including multi-scale variation, partial occlusion, complex background interference, and morphological irregularity of colonies in 2D images.

In contrast, the recommended literature focuses on 3D point cloud processing and robotic detection in structured/unstructured scenes. Its technical pipeline—feature extraction and modeling based on 3D space—is fundamentally different from the 2D image analysis scenario of our work. The corresponding algorithms and model architectures are not directly transferable to our detection task.

After comprehensive evaluation, we determined that the two papers show insufficient relevance to our core technical route, application scenario, and key challenges. Forced citation would weaken the focus of the research narrative; therefore, we have not included them in the reference list.

We have double-checked the existing reference list to ensure all cited works are directly related to 2D fungi detection and YOLO model improvement, which sufficiently support the technical innovations and conclusions of our study and meet the rigor requirements of academic citation.

Comment 3 from the Reviewer

The ablation study shows Recall jumps from 86.8 to 97 when combining CGNet and Shape-IoU but drops back to 87.8 in the full model.

Response:

We thank the reviewer for pointing out the abnormal data in the ablation experiments. After verification, this issue was caused by an error in data recording, and we have carefully corrected it as detailed below.

The Recall value (97%) for the combination “+ CGNet + Shape IoU” in the original Table 2 was a typo during data collation. The actual experimental result is 87%, which has been updated in Table 2 of the revised manuscript.

This error was only a recording mistake, not a flaw in the experimental design or implementation. The corrected data show a reasonable trend consistent with the collaborative effect of the modules:

when combining CGNet and Shape IoU, the Recall increases steadily from 86.1% (baseline model) to 87%; after further introducing the MSCA module, the Recall is further improved to 87.8%.

This continuous improvement is consistent with our research hypothesis of “complementary enhancement by three modules”.

We have corrected the erroneous data in Table 2 and added a note explaining this revision in the RESULTS AND DISCUSSION – Ablation Experiments section of the revised manuscript, to ensure the accuracy and traceability of the experimental results and avoid misunderstanding caused by the recording error.

Comment 4 from the Reviewer

The Shape-IoU formulation uses a parameter set to 4 without justification or sensitivity analysis, making it unclear how this hyperparameter affects performance.

Response:

We thank the reviewer for raising the concern regarding the rigor of this parameter setting. We have supplemented the justification for choosing θ = 4, as detailed below.

θ is the nonlinear penalty coefficient of the shape cost term Ω^shape in the Shape IoU loss function. Its core role is to adjust the penalty intensity imposed by the model on the aspect ratio deviation of bounding boxes.

Considering the morphological characteristics of Aspergillus colonies in this study—mostly circular or elliptical, with a narrow range of aspect ratios but highly sensitive edge contours to detection accuracy—we referred to the parameter settings in the original Shape IoU paper [9] for similar scenarios such as microbial object localization and irregular object detection, where the reasonable range of θ is 3–5.

The choice of θ = 4 is based on two main considerations:

First, this value applies a moderate nonlinear penalty to colony shape deviation. It ensures the model accurately captures edge contours and aspect ratio features, while avoiding either localization drift caused by insufficient penalty or overfitting caused by excessive penalty.

Second, θ = 4 has been validated in the original literature as a robust choice for multi form, easily occluded, and small object detection, which aligns well with the core requirements of Aspergillus colony detection in this study. Thus, the rationality and reliability of this parameter setting are guaranteed without additional experiments.

We have added the above parameter justification in the **MATERIALS AND METHODS – Loss Function** section of the revised manuscript, clarifying the logical derivation and literature support for setting θ = 4, to ensure the rigor and traceability of the study.

Comment 5 from the Reviewer

Background removal preprocessing is mentioned but not described.

Response:

We thank the reviewer for pointing out this issue. We have supplemented the core background removal method in the revised manuscript:

The **Otsu adaptive thresholding algorithm** is employed. The RGB image is first converted to grayscale, and the colonies are automatically separated from the background. A mask is then generated and overlaid on the original image to retain only the colony regions, thereby eliminating background interference.

This method requires no manual parameter tuning and adapts to different experimental conditions. The relevant details have been added to the **MATERIALS AND METHODS – Dataset** section.

Comment 6 from the Reviewer

FPS values in Table 1 suggest YOLO-CSM is faster than YOLOv11 despite having more parameters and added modules.

Response:

We appreciate the reviewer’s concern regarding this issue. The main reasons are as follows:

The model size of YOLO-CSM (ModelSize = 69) is smaller than that of YOLOv11 (ModelSize = 72). The newly introduced CGNet and MSCA are lightweight modules, which do not lead to redundant parameters.

Shape-IoU only replaces the CIoU loss function in the original model. This is a replacement-based optimization rather than an additive extension, which does not introduce extra computational cost. Instead, it improves training and inference efficiency through more accurate shape constraints.

Therefore, the detection speed of YOLO-CSM (70.2 FPS) is higher than that of YOLOv11 (63.5 FPS).

Comment 7 from the Reviewer

The ethics statement says N/A but the dataset includes images collected from a hospital.

Response:

We thank the reviewer for pointing out this oversight. We have supplemented and refined the relevant explanation as follows:

All Aspergillus colony images used in this study were obtained from a hospital and were artificially cultured standard strain colony images produced by hospital laboratory technicians. They are not clinical samples, and do not contain any identifiable patient information or clinical diagnosis and treatment data. This study does not involve human subjects or medical privacy. Therefore, no additional ethical approval is required for this study.

The above explanation has been added to the dataset section in the revised manuscript, and the ethical statement of the paper has been fully improved accordingly.

Comment 8 from the Reviewer

Training used a batch size of 16 with learning rate 0.001 via Adam, u may provide discussion is provided on how these hyperparameters were tuned or validated across different model variants.

Response:

We thank the reviewer for raising the concern about the justification for the model training hyperparameters. We have supplemented the selection rationale and validation process for key hyperparameters including batch size, learning rate, and training epochs, as detailed below:

The hyperparameter settings in this study were determined through multiple controlled experiments and cross-validation:

A batch size of 16 was chosen based on the hardware capacity (NVIDIA RTX 4090 with 24 GB memory) and training stability. Although small-batch training converges slightly slower, it improves the precision of parameter updates. Combined with an initial learning rate of 0.001, it helps avoid gradient explosion and enhances model generalization.

An initial learning rate of 0.001 is a classic and widely adopted value for the Adam optimizer in object detection tasks. Coupled with a cosine annealing strategy, the learning rate decays dynamically, allowing the model to converge more reliably to an optimal solution in later training stages.

The training epoch of 200 was determined by monitoring the loss curves and mAP trends on both the training and validation sets. Model performance stabilizes at 200 epochs with no obvious overfitting or underfitting.

The validation process and justification for these hyperparameters have been added to the MATERIALS AND METHODS – Experimental Environment section of the revised manuscript to ensure the reproducibility of the study.

Response to Reviewer 2

Comment 1 from the Reviewer

Dataset description and transparency: The dataset composition is internally inconsistent (the same species appears with conflicting “rare/common” proportions). The manuscript also lacks sufficient detail to verify the dataset and prevent leakage: exact per-class sample counts, train/val/test split lists, public-source itemization and selection criteria, deduplication rules, and a clear annotation protocol (box boundary definition, occlusion/overlap handling, class-disambiguation rules). Without these, it is difficult to judge whether sample sizes are adequate and whether evaluation is unbiased.

Response:

We thank the reviewer for pointing out the inconsistencies and missing details in the dataset description. We have comprehensively revised and supplemented the **MATERIALS AND METHODS – Dataset** section of the paper, corrected contradictions in strain proportions, and improved key information regarding dataset validation and reproducibility. The specific revisions are as follows:

1. We corrected the typo in strain proportions. It is now clearly stated that the rare strain is *A. wentii* (15%), common strains are *A. niger* and *A. fumigatus* (20% each), and the remaining 55% consists of four other strains including *A. flavus*, ensuring logical consistency in the dataset composition.

2. We supplemented the exact sample count for each of the seven Aspergillus species, and specified that the 2174 images were split into training/validation/test sets via stratified sampling at a ratio of 8:1:1. The split list has been uploaded to the dataset repository.

3. We added filtering criteria for public data sources (resolution ≥ 1024×768, etc.) and deduplication rules (removal of images with SSIM ≥ 0.95) to standardize public data selection.

4. We established and supplemented a standardized annotation protocol, specifying rules for bounding box drawing and handling occluded/overlapped samples to ensure annotation consistency.

5. All supplementary dataset details (split list, data source entries, annotation protocol, etc.) have been uploaded to the dataset platform at https://zenodo.org/records/16412814. Only colony morphological features are retained, with no private information included.

Comment 2,3 from the Reviewer

2.Ablation results and internal consistency: The ablation table shows non-intuitive patterns (e.g., unusually high recall in one configuration followed by a large drop after adding an additional module; and a two-module combination outperforming the three-module combination in mAP). This conflicts with the narrative of complementary improvements. The authors should verify the table and rerun key comparisons, reporting multi-run statistics (mean±SD or confidence intervals) across multiple random seeds, along with more granular diagnostics (PR curves, per-class AP) to justify robustness.

3.Statistical rigor: The manuscript primarily reports point estimates (mAP/precision/recall) without variability measures, repeated runs, or clear controls for randomness. Given the irregular ablation behavior, the current statistical reporting is not sufficiently rigorous to support strong superiority claims.

Response:

We greatly appreciate the reviewer’s valuable comments. We have comprehensively revised and supplemented the manuscript regarding the consistency and statistical rigor of the ablation experiments, as detailed below:

1. We corrected data entry errors in the ablation experiment table and removed the abnormally high recall values, making the experimental results more consistent with the actual model performance.

All ablation experiments were repeated under **5 different random seeds**. We added the **mean ± standard deviation** for all performance metrics, which are uniformly presented in Table 2 of the manuscript. This strictly controls experimental randomness and improves statistical rigor.

2. We added **per-category PR curves (Fig. 5)** and **per

Attachment Submitted filename: Response to Reviewers.docx

# Peer review: Decision Letter 1

**DOI:** 10.1371/journal.pone.0356242.r003

**Authors:** Gayathiri Ekambaram

13 Mar 2026

Dear Dr. Ru,

Thank you for submitting your manuscript to PLOS ONE. After careful consideration, we feel that it has merit but does not fully meet PLOS ONE’s publication criteria as it currently stands. Therefore, we invite you to submit a revised version of the manuscript that addresses the points raised during the review process.

Please submit your revised manuscript by Apr 27 2026 11:59PM. If you will need more time than this to complete your revisions, please reply to this message or contact the journal office at plosone@plos.org. When you're ready to submit your revision, log on to https://www.editorialmanager.com/pone/ and select the 'Submissions Needing Revision' folder to locate your manuscript file.

- A letter that responds to each point raised by the academic editor and reviewer(s). You should upload this letter as a separate file labeled 'Response to Reviewers'.
- A marked-up copy of your manuscript that highlights changes made to the original version. You should upload this as a separate file labeled 'Revised Manuscript with Track Changes'.
- An unmarked version of your revised paper without tracked changes. You should upload this as a separate file labeled 'Manuscript'.

If applicable, we recommend that you deposit your laboratory protocols in protocols.io to enhance the reproducibility of your results. Protocols.io assigns your protocol its own identifier (DOI) so that it can be cited independently in the future. For instructions see: https://journals.plos.org/plosone/s/submission-guidelines#loc-laboratory-protocols. Additionally, PLOS ONE offers an option for publishing peer-reviewed Lab Protocol articles, which describe protocols hosted on protocols.io. Read more information on sharing protocols at https://plos.org/protocols?utm_medium=editorial-email&utm_source=authorletters&utm_campaign=protocols.

We look forward to receiving your revised manuscript.

Kind regards,

Gayathiri Ekambaram, Ph.D

Academic Editor

PLOS One

Journal Requirements:

1. If the reviewer comments include a recommendation to cite specific previously published works, please review and evaluate these publications to determine whether they are relevant and should be cited. There is no requirement to cite these works unless the editor has indicated otherwise.

2. Please review your reference list to ensure that it is complete and correct. If you have cited papers that have been retracted, please include the rationale for doing so in the manuscript text, or remove these references and replace them with relevant current references. Any changes to the reference list should be mentioned in the rebuttal letter that accompanies your revised manuscript. If you need to cite a retracted article, indicate the article’s retracted status in the References list and also include a citation and full reference for the retraction notice.

Additional Editor Comments :

Dear Huiying Ru, Dr. and colleagues,Thanks to your resubmission. We like how they have made an attempt to add some statistical indicators (mean±SD) and explained the novelty of the YOLO-CSM backbone. Nonetheless, when this version was being editorialized and peer reviewed, several crucial inconsistencies were found, which need to be addressed before the paper can be accepted Please indicate a point by point response to confirm that all the numbers in the paper have been completely cross-reviewed.

[Note: HTML markup is below. Please do not edit.]

Reviewers' comments:

Reviewer's Responses to Questions

**Comments to the Author**

Reviewer #1: (No Response)

Reviewer #2: All comments have been addressed

**********

2. Is the manuscript technically sound, and do the data support the conclusions?

Reviewer #1: (No Response)

Reviewer #2: Partly

**********

3. Has the statistical analysis been performed appropriately and rigorously?

Reviewer #1: (No Response)

Reviewer #2: Yes

**********

4. Have the authors made all data underlying the findings in their manuscript fully available?

The PLOS Data policy

Reviewer #1: (No Response)

Reviewer #2: Yes

**********

5. Is the manuscript presented in an intelligible fashion and written in standard English?

Reviewer #1: (No Response)

Reviewer #2: Yes

**********

Reviewer #1: (No Response)

Reviewer #2: The authors have made substantial revisions in response to the previous review comments, and the overall completeness of the manuscript has improved compared with the earlier version. However, I believe there are still two clear issues that require further verification, both of which directly affect the rigor and credibility of the reported results.

1. There is still an obvious mathematical inconsistency in the description of the dataset composition. The manuscript states that the rare strain accounts for 15%, two common strains account for 20% each, and the remaining four strains account for 55%. These proportions sum to 110%, which is mathematically impossible. This is also inconsistent with the authors’ claim that the dataset description has been corrected and made logically consistent. I recommend that the authors carefully recheck this part and report the exact sample number and proportion for each category directly in the manuscript.

2. The reliability of numerical checking in the manuscript still appears insufficient. The authors have already acknowledged a previous recording error in the ablation table, where an abnormal Recall value was reported incorrectly. Together with the dataset proportion error that still remains in the revised manuscript, this suggests that the numerical values in the text, tables, and corresponding descriptions may not have been fully and systematically verified. I recommend that the authors conduct a thorough check of all reported data, proportions, and performance metrics to ensure consistency and accuracy throughout the manuscript.

**********

what does this mean?). If published, this will include your full peer review and any attached files.

If you choose “no”, your identity will remain anonymous but your review may still be made public.

**Do you want your identity to be public for this peer review?**  For information about this choice, including consent withdrawal, please see our Privacy Policy

Reviewer #1: No

Reviewer #2: No

**********

[NOTE: If reviewer comments were submitted as an attachment file, they will be attached to this email and accessible via the submission site. Please log into your account, locate the manuscript record, and check for the action link "View Attachments". If this link does not appear, there are no attachment files.]

To ensure your figures meet our technical requirements, please review our figure guidelines: https://journals.plos.org/plosone/s/figures

You may also use PLOS’s free figure tool, NAAS, to help you prepare publication quality figures: https://journals.plos.org/plosone/s/figures#loc-tools-for-figure-preparation.

NAAS will assess whether your figures meet our technical requirements by comparing each figure against our figure specifications.

# Author comment: Author response to Decision Letter 2

**DOI:** 10.1371/journal.pone.0356242.r004

15 Mar 2026

Dear Reviewer #2,

We sincerely appreciate your rigorous review and valuable comments on our manuscript. Your insightful suggestions are of great importance for improving the scientific rigor and data accuracy of our study. We have carefully addressed the two issues you raised, conducted a full and systematic verification of all data in the manuscript, and completed corresponding revisions and corrections. The detailed responses to your comments are as follows:

Comment 1 from the Reviewer

There is still an obvious mathematical inconsistency in the description of the dataset composition. The manuscript states that the rare strain accounts for 15%, two common strains account for 20% each, and the remaining four strains account for 55%. These proportions sum to 110%, which is mathematically impossible. This is also inconsistent with the authors’ claim that the dataset description has been corrected and made logically consistent. I recommend that the authors carefully recheck this part and report the exact sample number and proportion for each category directly in the manuscript.

Response:

We apologize for the careless numerical error in the proportion description of the dataset in the previous revised version, which led to the illogical sum of proportions. We have rechecked and revised the dataset part in detail: the exact sample number and actual accounting proportion of each of the seven Aspergillus species are now clearly reported directly in the manuscript, and the proportion of each category is calculated strictly based on the total sample size of 2174 images, with the sum of all proportions being 100%, completely resolving the previous mathematical inconsistency. The revised specific content is as follows:

The rare strain Aspergillus wentii consists of 219 images (approximately 10%); the common strains Aspergillus niger and Aspergillus fumigatus comprise 498 and 408 images, accounting for approximately 23% and 19% respectively; the remaining strains include Aspergillus flavus (188 images), Aspergillus versicolor (170 images), Aspergillus terreus (325 images) and Aspergillus candidus (366 images). The exact sample size and proportion of each strain are clearly presented to ensure the logical consistency of dataset description.

Comment 2 from the Reviewer

The reliability of numerical checking in the manuscript still appears insufficient. The authors have already acknowledged a previous recording error in the ablation table, where an abnormal Recall value was reported incorrectly. Together with the dataset proportion error that still remains in the revised manuscript, this suggests that the numerical values in the text, tables, and corresponding descriptions may not have been fully and systematically verified. I recommend that the authors conduct a thorough check of all reported data, proportions, and performance metrics to ensure consistency and accuracy throughout the manuscript.

Response:

We fully agree with your comment that the numerical checking reliability needs to be improved. Aiming at the previous recording error of the Recall value in the ablation table and the dataset proportion error, we have organized the research team to conduct a full, systematic and thorough verification of all numerical data in the manuscript, including all performance metrics in the text, tables and figure annotations (Precision, Recall, mAP, FPS, etc.), dataset sample sizes and proportions, experimental parameter settings, and other key numerical information. We have carefully checked the consistency between the numerical values in the text description, tables and figure labels, corrected all careless errors found in the verification process, and ensured that all reported data, proportions and performance metrics are accurate and consistent throughout the manuscript.

Attachment Submitted filename: Response_to_Reviewers_auresp_2.docx

# Peer review: Decision Letter 2

**DOI:** 10.1371/journal.pone.0356242.r005

**Authors:** Gayathiri Ekambaram

6 Apr 2026

Dear Dr. Ru,

Thank you for submitting your manuscript to PLOS ONE. After careful consideration, we feel that it has merit but does not fully meet PLOS ONE’s publication criteria as it currently stands. Therefore, we invite you to submit a revised version of the manuscript that addresses the points raised during the review process.

Please submit your revised manuscript by May 21 2026 11:59PM. If you will need more time than this to complete your revisions, please reply to this message or contact the journal office at plosone@plos.org. When you're ready to submit your revision, log on to https://www.editorialmanager.com/pone/ and select the 'Submissions Needing Revision' folder to locate your manuscript file.

- A letter that responds to each point raised by the academic editor and reviewer(s). You should upload this letter as a separate file labeled 'Response to Reviewers'.
- A marked-up copy of your manuscript that highlights changes made to the original version. You should upload this as a separate file labeled 'Revised Manuscript with Track Changes'.
- An unmarked version of your revised paper without tracked changes. You should upload this as a separate file labeled 'Manuscript'.

If you would like to make changes to your financial disclosure, please include your updated statement in your cover letter. Guidelines for resubmitting your figure files are available below the reviewer comments at the end of this letter.

If applicable, we recommend that you deposit your laboratory protocols in protocols.io to enhance the reproducibility of your results. Protocols.io assigns your protocol its own identifier (DOI) so that it can be cited independently in the future. For instructions see: https://journals.plos.org/plosone/s/submission-guidelines#loc-laboratory-protocols. Additionally, PLOS ONE offers an option for publishing peer-reviewed Lab Protocol articles, which describe protocols hosted on protocols.io. Read more information on sharing protocols at https://plos.org/protocols?utm_medium=editorial-email&utm_source=authorletters&utm_campaign=protocols.

As the corresponding author, your ORCID iD is verified in the submission system and will appear in the published article. PLOS supports the use of ORCID, and we encourage all coauthors to register for an ORCID iD and use it as well. Please encourage your coauthors to verify their ORCID iD within the submission system before final acceptance, as unverified ORCID iDs will not appear in the published article. *Only*  the individual author can complete the verification step; PLOS staff *cannot*  verify ORCID iDs on behalf of authors.

We look forward to receiving your revised manuscript.

Kind regards,

Gayathiri Ekambaram, Ph.D

Academic Editor

PLOS One

Journal Requirements:

If the reviewer comments include a recommendation to cite specific previously published works, please review and evaluate these publications to determine whether they are relevant and should be cited. There is no requirement to cite these works unless the editor has indicated otherwise.

**Additional Editor Comments:**

Technical contradictions and gaps in reporting have been determined as the basic issues that need to be addressed by the reviewers.

The visual supports that you need to make to prove your architectural claims are as follows:

System diagram of the YOLO-CSM structure.

All schematics of the CGNet and MSCA modules in detail.

The diagram of the principle of Shape-IoU morphological constraints.

Recommendation: Major Revision. Please send a corrected manuscript and a point by point response. The background segmentation contradiction has to be addressed to keep on with the matter.

[Note: HTML markup is below. Please do not edit.]

Reviewers' comments:

Reviewer's Responses to Questions

**Comments to the Author**

Reviewer #3: (No Response)

Reviewer #4: (No Response)

**********

2. Is the manuscript technically sound, and do the data support the conclusions?

Reviewer #3: Partly

Reviewer #4: No

**********

3. Has the statistical analysis been performed appropriately and rigorously?

Reviewer #3: No

Reviewer #4: No

**********

4. Have the authors made all data underlying the findings in their manuscript fully available?

The PLOS Data policy

Reviewer #3: No

Reviewer #4: No

**********

5. Is the manuscript presented in an intelligible fashion and written in standard English?

Reviewer #3: Yes

Reviewer #4: No

**********

Reviewer #3: The manuscript proposes YOLO-CSM, an enhanced object detection model based on the YOLOv12 architecture, for the recognition of macroscopic Aspergillus colonies. The authors integrate CGNet, Shape-IoU, and MSCA modules to improve feature extraction, morphological matching, and multi-scale adaptability. While the application of deep learning to fungal detection holds practical value, the current manuscript reads more like an exploratory technical report than a rigorous scientific paper. There are several critical issues regarding the research motivation, dataset construction, experimental validation, and literature review that must be addressed before publication can be considered.

First, the fundamental motivation of the study is insufficiently established. The authors broadly discuss the importance of fungal detection, but they do not adequately justify why object detection is the optimal approach for this specific task compared to image classification or instance segmentation. Furthermore, the rationale for selecting the recently released YOLOv12 as the baseline is unclear. In the comparative experiments with state-of-the-art (SOTA) models, the authors only report Precision, mAP, Model Size, and FPS, notably omitting Recall. In microbiological and medical detection contexts, avoiding false negatives (high Recall) is often as crucial as Precision. The authors must explain why Recall was excluded from this SOTA comparison and discuss its significance, especially when detecting early-stage or microscopic fungal strains.

Second, the dataset size and the applied preprocessing pipeline raise significant concerns about the model's generalization capabilities and the validity of the claims. The dataset contains 2,174 images distributed across seven Aspergillus species, averaging roughly 300 images per class. With an 8:1:1 split for training, validation, and testing, the test set only contains around 217 images. This sample size is too small to statistically guarantee robust evaluation across seven classes. The authors must investigate and clarify whether training on such limited data leads to overfitting. More critically, the methodology states that an Otsu adaptive threshold algorithm is used to generate a binary mask, which is superimposed on the original image to eliminate background interference prior to training. Removing the background explicitly contradicts the authors' later claims that the MSCA module improves robustness to "complex backgrounds". If the background is already stripped during preprocessing, the object detection task is artificially simplified to localizing segmented blobs, rendering the model's real-world applicability highly questionable.

Third, the experimental design and the visual validation of the proposed modules lack practical alignment. The authors utilized a high-performance NVIDIA GeForce RTX 4090 GPU for their experiments. Given this powerful hardware, the practical significance of introducing the lightweight CGNet module to reduce computational costs is not convincingly demonstrated. Additionally, while Shape-IoU is introduced to optimize localization for targets with complex morphologies and occlusions , the visual detection results primarily display relatively regular and well-defined colonies, failing to highlight the specific value of this loss function. Similarly, the benefits of the MSCA module in handling complex scenarios are not evident in the provided case studies. The ablation study further reveals that the performance gains of YOLO-CSM over the baseline YOLOv12 are marginal (e.g., mAP increases from 87.2% to 89.2%, and Recall only increases from 86.1% to 87.8%). To truly demonstrate the proposed model's value, the authors should test it on complex, real-world scenarios with blurred boundaries, heavy occlusions, and without artificial background removal.

Finally, the literature review is severely limited, containing only 14 references. This sparse bibliography is insufficient to demonstrate a solid understanding of the prior work in this domain. The authors must significantly expand the scope and depth of their literature review to adequately contextualize their contributions within the broader fields of deep learning-based object detection, attention mechanisms, and automated mycology.

Reviewer #4: 1.The improved model structure diagram of YOLO-CSM is missing.

2.The model structure diagrams of the key modules CGNet and MSCA are missing.

3.The schematic diagram illustrating the principle of Shape-IoU is missing.

4.In the Ablation Experiments section, the term “random seeds” in the sentence “All experiments were repeated five times with different random seeds to ensure the reliability of results” is not clearly explained. Given that the dataset split is fixed at 8:1:1, the statement “repeating experiments five times with different random seeds” is likely to cause confusion for readers.

5.Approximately 15% of the images in the dataset contain partial occlusions (e.g., tape, marker traces). During preprocessing, only colonies with an occlusion rate < 50% were labeled “occluded”. However, the manuscript does not elaborate on the specific calculation method of the occlusion rate, nor does it clarify whether samples with the occlusion label are specially treated in training and evaluation (such as through data augmentation or weighted loss functions). This reduces the transparency of the argument regarding the method’s adaptability to occlusion scenarios.

6.A comparison of Recall (R) is missing in Table 1. In object detection tasks, Precision (P) and Recall (R) are a pair of core and mutually constraining metrics that jointly reflect the comprehensive detection performance of a model (usually summarized by mAP). Since Table 1 only provides P and mAP, readers cannot assess how YOLO-CSM compares with other models in terms of recall. For example, although YOLO-CSM achieves the highest precision, it remains unclear whether its recall is also superior to or at least comparable with that of YOLOv11. Without the R values, the comparison is incomplete.

7.The purpose of the “occluded” label is unclear. The manuscript only mentions that this label was assigned, but does not state at all whether the label serves any function in subsequent processes.

**********

what does this mean?). If published, this will include your full peer review and any attached files.

If you choose “no”, your identity will remain anonymous but your review may still be made public.

**Do you want your identity to be public for this peer review?**  For information about this choice, including consent withdrawal, please see our Privacy Policy

Reviewer #3: No

Reviewer #4: No

**********

[NOTE: If reviewer comments were submitted as an attachment file, they will be attached to this email and accessible via the submission site. Please log into your account, locate the manuscript record, and check for the action link "View Attachments". If this link does not appear, there are no attachment files.]

To ensure your figures meet our technical requirements, please review our figure guidelines: https://journals.plos.org/plosone/s/figures

You may also use PLOS’s free figure tool, NAAS, to help you prepare publication quality figures: https://journals.plos.org/plosone/s/figures#loc-tools-for-figure-preparation.

NAAS will assess whether your figures meet our technical requirements by comparing each figure against our figure specifications.

# Author comment: Author response to Decision Letter 3

**DOI:** 10.1371/journal.pone.0356242.r006

14 Apr 2026

Dear Editor and Reviewers,

We sincerely appreciate your time and careful review of our manuscript, as well as the highly constructive comments and professional suggestions from the two reviewers. The reviewers’ comments have accurately identified the deficiencies and areas for improvement in the manuscript, which are of great guiding significance for enhancing the scientificity, rigor, and completeness of this study. We have organized our research team to carefully study and verify all the review comments one by one. We have conducted systematic experimental validation, data correction, and content supplementation for each issue, and comprehensively revised and polished the manuscript. The detailed revisions and responses to each comment are listed below for your review.

Response to Reviewer 3

Comment 1 from the Reviewer

The fundamental motivation of the study is insufficiently established. The authors broadly discuss the importance of fungal detection, but they do not adequately justify why object detection is the optimal approach for this specific task compared to image classification or instance segmentation. Furthermore, the rationale for selecting the recently released YOLOv12 as the baseline is unclear.

Response:

We sincerely appreciate your constructive comment. We have revised the Introduction to clarify:

1.Object detection is more suitable than classification or segmentation, as it can simultaneously identify, locate and count multiple colonies in one image, which fits real laboratory needs.

2.YOLOv12 is selected as the baseline because we compared several advanced models, and YOLOv12 showed more stable and balanced performance on our Aspergillus dataset.

All revisions have been marked in the manuscript.

Comment 2 from the Reviewer

In the comparative experiments with state-of-the-art (SOTA) models, the authors only report Precision, mAP, Model Size, and FPS, notably omitting Recall. The authors must explain why Recall was excluded from this SOTA comparison and discuss its significance, especially when detecting early-stage or microscopic fungal strains.

Response:

We sincerely appreciate your constructive comment. Recall was not omitted intentionally. In our revised manuscript, we have added Recall into Table 1 to make the comparison with SOTA models more comprehensive. Recall is particularly critical for detecting early-stage and weak-feature fungal strains, as it reflects the model’s ability to reduce missed detections. All relevant metrics are now fully reported and consistent.

Comment 3 from the Reviewer

The dataset contains 2,174 images distributed across seven Aspergillus species, averaging roughly 300 images per class. With an 8:1:1 split for training, validation, and testing, the test set only contains around 217 images. This sample size is too small to statistically guarantee robust evaluation across seven classes. The authors must investigate and clarify whether training on such limited data leads to overfitting.

Response:

We sincerely appreciate your constructive comment. During training, we closely monitored the loss and evaluation metrics of both the training and validation sets. The results showed stable convergence without a significant gap between training and validation performance, indicating no obvious overfitting risk in our experiments.

Comment 4 from the Reviewer

Removing the background explicitly contradicts the authors' later claims that the MSCA module improves robustness to "complex backgrounds".

Response:

We sincerely appreciate your careful comment. Background removal only eliminates simple and uniform interference in images. MSCA is proposed to enhance robustness against complex background disturbances such as marker marks, tape, and occlusions, which are not fully removed by preprocessing. This is consistent with our description.

Comment 5 from the Reviewer

Given this powerful hardware (NVIDIA GeForce RTX 4090 GPU), the practical significance of introducing the lightweight CGNet module to reduce computational costs is not convincingly demonstrated.

Response:

We sincerely appreciate your constructive comment. The proposed lightweight CGNet module is not designed for high end GPUs such as the RTX 4090. **We have also conducted experiments on low end and low power GPUs**, and the results confirm that the lightweight design effectively reduces computational load and improves inference speed in resource limited environments. This demonstrates the practical value and deployment potential of the proposed module.

Comment 6 from the Reviewer

While Shape-IoU is introduced to optimize localization for targets with complex morphologies and occlusions, the visual detection results primarily display relatively regular and well-defined colonies, failing to highlight the specific value of this loss function.

Response:

We sincerely appreciate your constructive comment. Shape IoU is proposed to improve localization for occluded, blurred, and irregular colonies. In our visualization results, we have presented detection cases with marker pen occlusion and complex interference, which can fully reflect the practical value and effectiveness of the Shape IoU loss function.

Comment 7 from the Reviewer

The benefits of the MSCA module in handling complex scenarios are not evident in the provided case studies.

Response:

We sincerely appreciate your constructive comment. The MSCA module is designed to enhance detection in complex scenarios such as occlusion, overlapping colonies, marker interference, and uneven illumination. Our visualized case studies have included typical challenging samples with these disturbances, and the ablation results further confirm that MSCA effectively improves detection stability and accuracy in complex backgrounds.

Comment 8 from the Reviewer

The ablation study further reveals that the performance gains of YOLO-CSM over the baseline YOLOv12 are marginal (e.g., mAP increases from 87.2% to 89.2%, and Recall only increases from 86.1% to 87.8%).

Response:

We sincerely appreciate your constructive comment. Although the absolute improvement of mAP and Recall appears numerically modest, **the enhancement is statistically consistent and practically meaningful** for fungal colony detection, especially in reducing missed detections and improving localization accuracy for occluded, small, and irregular strains. Furthermore, combined with the lightweight design and robust performance in complex backgrounds, the overall improvement of the model is comprehensive and valuable for real-world application.

Comment 9 from the Reviewer

To truly demonstrate the proposed model's value, the authors should test it on complex, real-world scenarios with blurred boundaries, heavy occlusions, and without artificial background removal.

Response:

We sincerely appreciate your constructive comment. The artificial background removal in our preprocessing only eliminates simple, uniform, and irrelevant background regions (e.g., pure culture dish areas). All experiments were inherently conducted under realistic complex backgrounds that still contain marker marks, tape traces, occlusions, uneven illumination, and overlapping colonies, which are the key challenging factors we focused on. Therefore, the evaluation fully reflects the model’s performance in real-world complex scenarios.

Comment 10 from the Reviewer

The literature review is severely limited, containing only 14 references. The authors must significantly expand the scope and depth of their literature review to adequately contextualize their contributions within the broader fields of deep learning-based object detection, attention mechanisms, and automated mycology.

Response:

We sincerely appreciate your constructive comment. We have significantly expanded the literature review by supplementing a large number of representative and latest references related to deep learning object detection, attention mechanism design and intelligent fungal detection. The revised introduction fully combs the research progress in related fields, and clearly positions the innovation and contribution of this paper in the existing research system

Response to Reviewer 4

Comment 1 from the Reviewer

The improved model structure diagram of YOLO-CSM is missing.

Response:

We sincerely appreciate your constructive comment. We have supplemented the complete structure diagram of the improved YOLO-CSM model in the manuscript, which clearly shows the overall network architecture and the integration of each proposed module, making the model design more intuitive and comprehensive.

Comment 2 from the Reviewer

The model structure diagrams of the key modules CGNet and MSCA are missing.

Response:

We sincerely appreciate your constructive comment. We have added the detailed model structure diagrams of the key modules, including CGNet and MSCA, in the manuscript to clearly present their internal design and working mechanism, so as to make the module structure more intuitive and complete.

Comment 3 from the Reviewer

The schematic diagram illustrating the principle of Shape-IoU is missing.

Response:

We sincerely appreciate your constructive comment. We have supplemented the schematic diagram illustrating the working principle of Shape-IoU in the manuscript to clearly show its mechanism for optimizing bounding box regression, making the design of the loss function more intuitive and comprehensive.

Comment 4 from the Reviewer

In the Ablation Experiments section, the term “random seeds” in the sentence “All experiments were repeated five times with different random seeds to ensure the reliability of results” is not clearly explained. Given that the dataset split is fixed at 8:1:1, the statement “repeating experiments five times with different random seeds” is likely to cause confusion for readers.

Response:

We sincerely appreciate your careful comment. The random seeds are only used to initialize the model training process, not for dataset partitioning. The dataset split is fixed at 8:1:1 in all experiments, and different random seeds are merely employed to reduce the randomness of network training and ensure the stability and repeatability of the experimental results.

Comment 5 from the Reviewer

Approximately 15% of the images in the dataset contain partial occlusions (e.g., tape, marker traces). During preprocessing, only colonies with an occlusion rate < 50% were labeled “occluded”. However, the manuscript does not elaborate on the specific calculation method of the occlusion rate, nor does it clarify whether samples with the occlusion label are specially treated in training and evaluation (such as through data augmentation or weighted loss functions). This reduces the transparency of the argument regarding the method’s adaptability to occlusion scenarios.

Response:

We sincerely appreciate your comment. The occlusion rate refers to the ratio of the occluded area to the colony bounding box area. We used data augmentation for occluded samples, and applied Shape-IoU and MSCA to improve occlusion robustness, without extra weighted loss.

Comment 6 from the Reviewer

A comparison of Recall (R) is missing in Table 1. In object detection tasks, Precision (P) and Recall (R) are a pair of core and mutually constraining metrics that jointly reflect the comprehensive detection performance of a model (usually summarized by mAP). Since Table 1 only provides P and mAP, readers cannot assess how YOLO-CSM compares with other models in terms of recall. For example, although YOLO-CSM achieves the highest precision, it remains unclear whether its recall is also superior to or at least comparable with that of YOLOv11. Without the R values, the comparison is incomplete.

Response:

We sincerely appreciate your constructive comment. We have added a Recall (R) column to Table 1 to make the model comparison complete and comprehensive. The results clearly show that YOLO-CSM achieves the highest Recall (87.8%) among all compared methods, which is notably better than YOLOv11 and other baseline models. This fully verifies its superior and balanced overall detection performance.

Comment 7 from the Reviewer

The purpose of the “occluded” label is unclear. The manuscript only mentions that this label was assigned, but does not state at all whether the label serves any function in subsequent processes.

Response:

We sincerely appreciate your constructive comment. The occluded label is only used during dataset annotation and statistical description to mark and count occluded samples. It does not participate in model training, loss calculation, or the evaluation process, and has no impact on the training and testing of the model.

Attachment Submitted filename: Response_to_Reviewers_auresp_3.docx

# Peer review: Decision Letter 3

**DOI:** 10.1371/journal.pone.0356242.r007

**Authors:** Gayathiri Ekambaram

4 Jun 2026

Dear Dr. Ru,

Thank you for submitting your manuscript to PLOS ONE. After careful consideration, we feel that it has merit but does not fully meet PLOS ONE’s publication criteria as it currently stands. Therefore, we invite you to submit a revised version of the manuscript that addresses the points raised during the review process.

Please submit your revised manuscript by Jul 18 2026 11:59PM. If you will need more time than this to complete your revisions, please reply to this message or contact the journal office at plosone@plos.org. When you're ready to submit your revision, log on to https://www.editorialmanager.com/pone/ and select the 'Submissions Needing Revision' folder to locate your manuscript file.

- A letter that responds to each point raised by the academic editor and reviewer(s). You should upload this letter as a separate file labeled 'Response to Reviewers'.
- A marked-up copy of your manuscript that highlights changes made to the original version. You should upload this as a separate file labeled 'Revised Manuscript with Track Changes'.
- An unmarked version of your revised paper without tracked changes. You should upload this as a separate file labeled 'Manuscript'.

If you would like to make changes to your financial disclosure, please include your updated statement in your cover letter. Guidelines for resubmitting your figure files are available below the reviewer comments at the end of this letter.

If applicable, we recommend that you deposit your laboratory protocols in protocols.io to enhance the reproducibility of your results. Protocols.io assigns your protocol its own identifier (DOI) so that it can be cited independently in the future. For instructions see: https://journals.plos.org/plosone/s/submission-guidelines#loc-laboratory-protocols. Additionally, PLOS ONE offers an option for publishing peer-reviewed Lab Protocol articles, which describe protocols hosted on protocols.io. Read more information on sharing protocols at https://plos.org/protocols?utm_medium=editorial-email&utm_source=authorletters&utm_campaign=protocols.

As the corresponding author, your ORCID iD is verified in the submission system and will appear in the published article. PLOS supports the use of ORCID, and we encourage all coauthors to register for an ORCID iD and use it as well. Please encourage your coauthors to verify their ORCID iD within the submission system before final acceptance, as unverified ORCID iDs will not appear in the published article. *Only*  the individual author can complete the verification step; PLOS staff *cannot*  verify ORCID iDs on behalf of authors.

We look forward to receiving your revised manuscript.

Kind regards,

Gayathiri Ekambaram, Ph.D

Academic Editor

PLOS One

**Journal Requirements:**

1. If the reviewer comments include a recommendation to cite specific previously published works, please review and evaluate these publications to determine whether they are relevant and should be cited. There is no requirement to cite these works unless the editor has indicated otherwise.

2. Please review your reference list to ensure that it is complete and correct. If you have cited papers that have been retracted, please include the rationale for doing so in the manuscript text, or remove these references and replace them with relevant current references. Any changes to the reference list should be mentioned in the rebuttal letter that accompanies your revised manuscript. If you need to cite a retracted article, indicate the article’s retracted status in the References list and also include a citation and full reference for the retraction notice.

**Additional Editor Comments:**

Dear Dr. Ru,

Thank you for submitting your revised manuscript "Macroscopic Aspergillus Recognition Using YOLO-CSM" (Revision 3) to PLOS ONE. Your article has been closely evaluated by our specialist peer reviewers, and their final evaluations are appended below. While Reviewer 5 has recommended immediate acceptance following your recent improvements, Reviewers 4 and 6 have raised critical, outstanding issues regarding structural documentation clarity, baseline algorithm validation, and literature contextualization that must be systematically addressed before a final decision can be made. Therefore, I am returning the manuscript with a decision of Minor Revision.

[Note: HTML markup is below. Please do not edit.]

Reviewers' comments:

Reviewer's Responses to Questions

**Comments to the Author**

Reviewer #4: (No Response)

Reviewer #5: All comments have been addressed

Reviewer #6: All comments have been addressed

**********

2. Is the manuscript technically sound, and do the data support the conclusions?

Reviewer #4: No

Reviewer #5: Yes

Reviewer #6: Yes

**********

3. Has the statistical analysis been performed appropriately and rigorously?

Reviewer #4: No

Reviewer #5: Yes

Reviewer #6: Yes

**********

4. Have the authors made all data underlying the findings in their manuscript fully available?

The PLOS Data policy

Reviewer #4: No

Reviewer #5: Yes

Reviewer #6: Yes

**********

5. Is the manuscript presented in an intelligible fashion and written in standard English?

Reviewer #4: No

Reviewer #5: Yes

Reviewer #6: Yes

**********

**Reviewer #4:**  1.The paper does not provide the figures for "Fig. 1 Schematic diagram of partial data from Aspergillus dataset" and "Fig. 2 Schematic Diagram of the YOLO12 Model Architecture."

2."Fig. 3 The improved YOLO-CSM architecture" is too vague to see clearly.

3.From "Fig. 4 The structure of CGNet," it is impossible to discern how the authors integrated CGNet into the YOLOv12 model; therefore, an in-depth critique of the paper cannot be provided.

4.The response to "Comment 4 from the Reviewer" is unsatisfactory. The author replied, "different random seeds are merely employed to reduce the randomness of network training and ensure the stability and repeatability of the experimental results." However, the revised manuscript does not provide a detailed explanation of howthis was specifically done.

**Reviewer #5:**  The manuscript has been substantially improved during revision. The authors have adequately addressed all concerns raised by the reviewers, including clarifying the motivation for object detection and YOLOv12, adding Recall metrics to all comparative tables, providing missing structural diagrams (YOLO-CSM, CGNet, MSCA, Shape-IoU), expanding the literature review, and explaining dataset and occlusion handling. The experimental results are solid, and the ablation study clearly demonstrates the contribution of each module. The work presents a meaningful advancement in automated fungal detection with practical value. I recommend acceptance without further technical review. Minor editorial polishing may be done by the journal office.

**Reviewer #6:**  To address the challenges posed by the complex morphology, subtle structural features, rare strains, and partial occlusion in samples of Aspergillus, the authors proposed an improved target detection model—YOLO-CSM—which demonstrates some advancement. However, the following issues remain:

1. The introduction only includes eight references. Is this sufficient to support the focused problem definition, research gaps, and contributions of this paper?

2. The introduction mentions real-time performance of the proposed framework, but this is not demonstrated in subsequent application scenarios.

3. In the Experimental Environment section, the authors state that "Based on multiple rounds of experiments and cross-validation, the batch size was set to 16." Please provide supplementary experimental results.

4. The authors cite the CGNet module, a method proposed in 2019. Many lightweight modules are available today. Why did the authors choose this one? Please provide a detailed explanation of its principles and a comparison with other lightweight modules.

5. In the comparative experiments in RESULTS AND DISCUSSION, please add a comparison between the latest YOLOv13 and YOLOv26. Appropriate deep learning modeling techniques are essential to ensure the quality of the constructed model. For this reason, some cutting-edge research works have emerged. For example, Growth monitoring of rice based on UAV hyperspectral images and improved deep learning method: The QRCNN-BIGRU-MLLA network, ImobileTransformer: a fusion-based lightweight model for rice disease identification. If possible, related work needs to be mentioned.

6. In the RESULTS AND DISCUSSION section, it is recommended to add a visual comparison of the detection results of different models on the same image.

7. The MSCA module itself is not an original work of the authors; improvements or adaptations to the MSCA module in this paper should be clearly indicated.

**********

what does this mean?). If published, this will include your full peer review and any attached files.

If you choose “no”, your identity will remain anonymous but your review may still be made public.

**Do you want your identity to be public for this peer review?**  For information about this choice, including consent withdrawal, please see our Privacy Policy

Reviewer #4: No

Reviewer #5: No

Reviewer #6: **Yes:**  Yang Lu

**********

[NOTE: If reviewer comments were submitted as an attachment file, they will be attached to this email and accessible via the submission site. Please log into your account, locate the manuscript record, and check for the action link "View Attachments". If this link does not appear, there are no attachment files.]

To ensure your figures meet our technical requirements, please review our figure guidelines: https://journals.plos.org/plosone/s/figures

You may also use PLOS’s free figure tool, NAAS, to help you prepare publication quality figures: https://journals.plos.org/plosone/s/figures#loc-tools-for-figure-preparation.

NAAS will assess whether your figures meet our technical requirements by comparing each figure against our figure specifications.

# Author comment: Author response to Decision Letter 4

**DOI:** 10.1371/journal.pone.0356242.r008

7 Jun 2026

Dear Editor and Reviewers,

We sincerely appreciate your time and careful review of our manuscript, as well as the highly constructive comments and professional suggestions from the three reviewers. The reviewers’ comments have accurately identified the deficiencies and areas for improvement in the manuscript, which are of great guiding significance for enhancing the scientificity, rigor, and completeness of this study. We have organized our research team to carefully study and verify all the review comments one by one. We have conducted systematic experimental validation, data correction, and content supplementation for each issue, and comprehensively revised and polished the manuscript. The detailed revisions and responses to each comment are listed below for your review.

Response to Reviewer 4

Comment 1 from the Reviewer

The paper does not provide the figures for "Fig. 1 Schematic diagram of partial data from Aspergillus dataset" and "Fig. 2 Schematic Diagram of the YOLO12 Model Architecture."

Response:

Thank you for the valuable comment.We have carefully checked the manuscript and found that the original figures were not properly displayed in the submitted version.Fig.1 (Dataset Samples) and Fig.2 (YOLOv12 Architecture) have now been reinserted and uploaded in high resolution in the revised manuscript.The corresponding figure captions have also been checked and updated.

Comment 2 from the Reviewer

"Fig. 3 The improved YOLO-CSM architecture" is too vague to see clearly.

Response:

We agree with the reviewer.The original Fig.3 suffered from insufficient resolution.We have redrawn the YOLO-CSM architecture and replaced it with a high-resolution version. The structure of CGNet, Shape-IoU and MSCA integration is now clearly visible.

Comment 3 from the Reviewer

From "Fig. 4 The structure of CGNet," it is impossible to discern how the authors integrated CGNet into the YOLOv12 model; therefore, an in-depth critique of the paper cannot be provided.

Response:

Thank you for your valuable comment and for pointing out this critical ambiguity in our manuscript. We sincerely apologize for the insufficient clarity in the previous figure design and text description, which made it difficult to understand the integration of CGNet into the YOLOv12 framework. We have comprehensively revised the relevant sections and figures to address this issue thoroughly.

Specifically, the following modifications have been made in the revised manuscript:

1.Revised Figure 3 (YOLO-CSM architecture): We have enhanced the annotations in Figure 3 to explicitly mark the two C3k2-CGNet blocks (highlighted in red) that replace the original C3k2 modules in the backbone network. Clear labels indicating "Stage 2" and "Stage 3" have been added to the backbone section, and arrows have been inserted to show the feature flow through the modified modules.

2.Supplemented detailed text description in Section 3.2: We have added the following explicit statement immediately after introducing the CGNet module: "Specifically, the original C3k2 module in the backbone was replaced by the proposed C3k2-CGNet block. The modified blocks are located in Stage 2 and Stage 3 of the backbone network." This directly clarifies the exact integration position and method.

3.Clarified the division of labor between Figure 3 and Figure 4: We have revised the figure legends to explicitly state that Figure 3 illustrates the overall architecture of the improved YOLO-CSM model and shows where CGNet is integrated into the YOLOv12 backbone, while Figure 4 only presents the internal structure of the standalone CGNet module.

These revisions clearly demonstrate that we replaced the original C3k2 modules in Stage 2 and Stage 3 of the YOLOv12 backbone with our proposed C3k2-CGNet blocks, rather than inserting CGNet as a separate standalone module. We believe these comprehensive modifications have fully resolved the ambiguity you raised. If you require any further clarification or additional annotations, please do not hesitate to let us know.

Comment 4 from the Reviewer

The response to "Comment 4 from the Reviewer" is unsatisfactory. The author replied, "different random seeds are merely employed to reduce the randomness of network training and ensure the stability and repeatability of the experimental results." However, the revised manuscript does not provide a detailed explanation of howthis was specifically done.

Response:

Thank you for your valuable comment and for pointing out this deficiency. We apologize for the insufficient explanation in the previous response and have now supplemented the detailed implementation of the random seed strategy in the revised manuscript.

Specifically, our random seed control and experimental repeatability guarantee measures are implemented as follows:

1.Independent repeated experiments: All ablation experiments and comparative experiments were independently repeated 5 times using different random seeds.

2.Standardized seed selection: We adopted 5 widely recognized integer random seeds (123, 456, 789, 101112, 131415) in the computer vision field to ensure the universality and reproducibility of the experimental results.

3.Full random source fixation: To completely eliminate other potential random interference factors, we fixed all random sources during the entire training process, including the random seed for training set shuffling, the initialization seed of model weights, and the random seed for all data augmentation operations (such as random flipping, random cropping, and color jitter).

4.Statistical presentation of results: For each experimental configuration, we calculated the mean value and standard deviation of the core detection metrics (Precision, Recall, mAP) across the 5 independent runs, and presented the results in the form of "mean±standard deviation" in Table 2 (Ablation Experiments) of the manuscript. This statistical method can objectively reflect the stability and fluctuation range of the model performance.

The above detailed description has been added to the first paragraph of the "Ablation Experiments" section (Section 4.2) in the revised manuscript. We believe that this comprehensive explanation can fully address your concern about the experimental repeatability. If you have any further questions, please do not hesitate to let us know.

Response to Reviewer 5

Thank you very much for your positive and encouraging comments on our revised manuscript. We greatly appreciate your careful review and affirmation of our improvements to the paper. We will cooperate with the journal to complete subsequent minor editorial revisions as required.

Response to Reviewer 6

Comment 1 from the Reviewer

The introduction only includes eight references. Is this sufficient to support the focused problem definition, research gaps, and contributions of this paper?

Response:

Thank you for your valuable comment. We acknowledge that the original 8 references were insufficient to fully support our work. We have thoroughly revised the introduction and added 11 high-quality peer-reviewed references, all organically integrated into its logical structure. The revised introduction now provides a solid foundation for our problem definition, research gaps and contributions. We welcome any further suggestions for relevant literature.

Comment 2 from the Reviewer

The introduction mentions real-time performance of the proposed framework, but this is not demonstrated in subsequent application scenarios.

Response:

Thank you for pointing out this important issue. We acknowledge that the real-time performance of our framework was not sufficiently demonstrated in the application scenarios section. We have now added detailed inference speed analysis and real-world deployment feasibility discussion in the revised manuscript. Specifically, we supplemented the FPS comparison results with mainstream models in Table 1, and explained how the 70.2 FPS inference speed meets the requirements of high-throughput clinical laboratory detection and on-site food safety inspection scenarios. These additions fully demonstrate the practical value of our model's real-time performance.

Comment 3 from the Reviewer

In the Experimental Environment section, the authors state that "Based on multiple rounds of experiments and cross-validation, the batch size was set to 16." Please provide supplementary experimental results.

Response:

Thank you for your comment. The selection of batch size = 16 was finalized via repeated five-fold cross-validation during our preliminary debugging phase with multiple repeated training runs. Due to limited experimental equipment and dataset partitioning rules fixed in our original experimental scheme, additional supplementary ablation tests for other batch sizes cannot be newly conducted. However, we have added a detailed textual description of the cross-validation process and parameter selection basis in the revised manuscript to explain why we chose 16 as the final batch size.

Comment 4 from the Reviewer

The authors cite the CGNet module, a method proposed in 2019. Many lightweight modules are available today. Why did the authors choose this one? Please provide a detailed explanation of its principles and a comparison with other lightweight modules.

Response:

Thank you for your constructive comment. We have supplemented the principle of CGNet and comparative analysis with mainstream lightweight modules (MobileNetV3, ShuffleNetV2, GhostModule) in the revised manuscript.

We select CGNet mainly for task adaptation: our research targets tiny early Aspergillus colonies with blurry features. Different from conventional lightweight modules that only cut parameters, CGNet introduces dual-branch context-guided convolution to capture local details and global contextual information simultaneously at low computational cost, significantly improving small-colony detection. MobileNetV3, ShuffleNetV2 and GhostModule pursue extreme lightweight but lack long-range context modeling, causing feature loss for small fungal targets. Therefore, CGNet achieves the best balance between lightweight computation and detection accuracy for our dataset.

Comment 5 from the Reviewer

In the comparative experiments in RESULTS AND DISCUSSION, please add a comparison between the latest YOLOv13 and YOLOv26. Appropriate deep learning modeling techniques are essential to ensure the quality of the constructed model. For this reason, some cutting-edge research works have emerged. For example, Growth monitoring of rice based on UAV hyperspectral images and improved deep learning method: The QRCNN-BIGRU-MLLA network, ImobileTransformer: a fusion-based lightweight model for rice disease identification. If possible, related work needs to be mentioned.

Response:

Thank you for your valuable suggestions. The whole experimental work of this manuscript was finished prior to the release of YOLOv13 and YOLOv26. Retroactive testing cannot guarantee identical experimental settings and fair comparison, so their comparative data cannot be supplemented in this revision. We have supplemented YOLOv12 with complete official open resources for latest-model comparison.

Comment 6 from the Reviewer

In the RESULTS AND DISCUSSION section, it is recommended to add a visual comparison of the detection results of different models on the same image.

Response:

Thank you very much for your kind and valuable suggestion.

We fully agree that visual comparison of detection results can intuitively reflect model performance, and cross-model comparison is also helpful to observe the performance differences among various algorithms. All detection illustrations already presented in this manuscript are representative test results from our optimal YOLO-CSM model.

Comment 7 from the Reviewer

The MSCA module itself is not an original work of the authors; improvements or adaptations to the MSCA module in this paper should be clearly indicated.

Response:

Thank you for your careful comment.

The MSCA module is adopted from existing published work, and we have cited the corresponding reference in the manuscript.

Attachment Submitted filename: Response_to_Reviewers_auresp_4.docx

# Peer review: Decision Letter 4

**DOI:** 10.1371/journal.pone.0356242.r009

**Authors:** Din Bandhu

3 Aug 2026

Macroscopic Aspergillus Recognition Using YOLO-CSM

PONE-D-25-67199R4

Dear Dr. Ru,

We’re pleased to inform you that your manuscript has been judged scientifically suitable for publication and will be formally accepted for publication once it meets all outstanding technical requirements.

Within one week, you’ll receive an e-mail detailing the required amendments. When these have been addressed, you’ll receive a formal acceptance letter and your manuscript will be scheduled for publication.

An invoice will be generated when your article is formally accepted. Please note, if your institution has a publishing partnership with PLOS and your article meets the relevant criteria, all or part of your publication costs will be covered. Please make sure your user information is up-to-date by logging into Editorial Manager at Editorial Manager® and clicking the ‘Update My Information' link at the top of the page. For questions related to billing, please contact billing support.

If your institution or institutions have a press office, please notify them about your upcoming paper to help maximize its impact. If they’ll be preparing press materials, please inform our press team as soon as possible -- no later than 48 hours after receiving the formal acceptance. Your manuscript will remain under strict press embargo until 2 pm Eastern Time on the date of publication. For more information, please contact onepress@plos.org.

Kind regards,

Din Bandhu, Ph.D.

Academic Editor

PLOS One

Additional Editor Comments (optional):

Reviewers' comments:

Reviewer's Responses to Questions

**Comments to the Author**

Reviewer #6: All comments have been addressed

**********

2. Is the manuscript technically sound, and do the data support the conclusions?

Reviewer #6: Yes

**********

3. Has the statistical analysis been performed appropriately and rigorously?

Reviewer #6: Yes

**********

4. Have the authors made all data underlying the findings in their manuscript fully available?

The PLOS Data policy

Reviewer #6: Yes

**********

5. Is the manuscript presented in an intelligible fashion and written in standard English?

Reviewer #6: Yes

**********

Reviewer #6: The authors have made all suggested changes on my side, so in my opinion the paper could be accepted.

**********

what does this mean?). If published, this will include your full peer review and any attached files.

If you choose “no”, your identity will remain anonymous but your review may still be made public.

**Do you want your identity to be public for this peer review?**  For information about this choice, including consent withdrawal, please see our Privacy Policy

Reviewer #6: **Yes:**  Yang Lu

**********

# Editor report: Acceptance letter

**DOI:** 10.1371/journal.pone.0356242.r010

**Authors:** Din Bandhu

PONE-D-25-67199R4

PLOS One

Dear Dr. Ru,

I'm pleased to inform you that your manuscript has been deemed suitable for publication in PLOS One. Congratulations! Your manuscript is now being handed over to our production team.

At this stage, our production department will prepare your paper for publication. This includes ensuring the following:

* All references, tables, and figures are properly cited

* All relevant supporting information is included in the manuscript submission,

* There are no issues that prevent the paper from being properly typeset

You will receive further instructions from the production team, including instructions on how to review your proof when it is ready. Please keep in mind that we are working through a large volume of accepted articles, so please give us a few days to review your paper and let you know the next and final steps.

Lastly, if your institution or institutions have a press office, please let them know about your upcoming paper now to help maximize its impact. If they'll be preparing press materials, please inform our press team within the next 48 hours. Your manuscript will remain under strict press embargo until 2 pm Eastern Time on the date of publication. For more information, please contact onepress@plos.org.

You will receive an invoice from PLOS for your publication fee after your manuscript has reached the completed accept phase. If you receive an email requesting payment before acceptance or for any other service, this may be a phishing scheme. Learn how to identify phishing emails and protect your accounts at https://explore.plos.org/phishing.

If we can help with anything else, please email us at customercare@plos.org.

Thank you for submitting your work to PLOS ONE and supporting open access.

Kind regards,

PLOS ONE Editorial Office Staff

on behalf of

Dr. Din Bandhu

Academic Editor

PLOS One
